# Music-Based Interventions in Advanced Cancer Palliative Care: A Systematic Review and Meta-Analysis of Randomized Controlled Trials Identified Within the Broader Field of Art-Based Interventions

**DOI:** 10.3390/jcm15187057

**Published:** 2026-09-11

**Authors:** Agata Śmiłowska, Magdalena Stania, Małgorzata Gajda, Justyna Niesporek, Anna Polak, Jolanta Grabowska-Markowska, Agnieszka Opala-Berdzik

**Affiliations:** 1 Department of Clinical Physiotherapy, Academy of Physical Education in Katowice, ul. Mikołowska 72B, 40-065 Katowice, Poland; 2 Hospice Cordis Association, ul. Teofila Ociepki 2, 40-413 Katowice, Poland; sekretariat@hospicjumcordis.pl; 3 Department of Theoretical and Practical Basics of Physiotherapy, Institute of Sport Sciences, Academy of Physical Education in Katowice, ul. Mikołowska 72B, 40-065 Katowice, Poland; m.stania@awf.katowice.pl; 4 Department of Physiotherapy in Internal Diseases, Academy of Physical Education in Katowice, ul. Mikołowska 72B, 40-065 Katowice, Poland; m.engelmann@awf.katowice.pl (M.G.); j.niesporek@awf.katowice.pl (J.N.); 5 Department of Clinical Physiotherapy, Institute of Physiotherapy and Health Sciences, Institute of Sport Sciences, Academy of Physical Education in Katowice, ul. Mikołowska 72B, 40-065 Katowice, Poland; a.polak@awf.katowice.pl; 6 Department of Clinical Physiotherapy, Institute of Physiotherapy and Health Sciences, Academy of Physical Education in Katowice, ul. Mikołowska 72B, 40-065 Katowice, Poland; a.opala-berdzik@awf.katowice.pl

**Keywords:** art therapy, music therapy, palliative care, neoplasms, pain management, systematic review, meta-analysis

## Abstract

**Background:** Art-based interventions, including music therapy, are increasingly used in palliative care to support symptom management and improve quality of life in patients with advanced cancer. This systematic review and meta-analysis aimed to evaluate their efficacy in adults with advanced cancer receiving palliative care. **Methods:** The review protocol was registered in PROSPERO (CRD42025636049). A systematic search of PubMed, MEDLINE (Ovid), and Embase was conducted from database inception to 17 December 2024 (PubMed), 21 December 2024 (MEDLINE (Ovid)), and 10 January 2025 (Embase), and was updated in February 2026. Additional searches were conducted in ClinicalTrials.gov, WHO ICTRP, Cochrane CENTRAL, and Google Scholar in August 2026. Studies comparing art-based interventions with standard care or active control interventions were included. Risk of bias was assessed using the Cochrane RoB 2 tool, and certainty of evidence using the GRADE approach. **Results:** Eleven reports with 9 RCTs (involving 675 patients) met the inclusion criteria, all evaluating music-based interventions. Music-based interventions in addition to routine palliative care were associated with a statistically significant reduction in pain intensity compared with control conditions (SMD = −0.51; 95%CI: −0.98 to −0.03; *p* = 0.04; k = 3; *n* =224), although high between-study heterogeneity was observed (I^2^ = 67.69%). They were also associated with higher quality-of-life scores (SMD = 0.92; 95%CI: 0.59 to 1.24; *p* < 0.0001; k = 2; *n* = 168; I^2^ = 0%); however, the certainty of evidence was very low, making this effect highly uncertain. **Conclusions:** Evidence for music-based interventions remains very uncertain, precluding firm conclusions regarding clinical benefit. Further high-quality trials, including other forms of art-based interventions, are needed.

## 1. Introduction

Patients with advanced cancer receiving palliative care experience profound, multidimensional suffering that substantially affects daily functioning and perceived quality of life [1,2,3]. In palliative care literature, this complex experience is conceptualized within the framework of total pain, proposed by Cicely Saunders, which describes suffering as the interaction among physical, psychological, social, and spiritual components [4]. As the disease progresses, somatic symptoms such as pain, fatigue, dyspnea, and sleep disturbances frequently co-occur with anxiety, depression, stress, loss of control, social difficulties, and existential distress, forming a comprehensive illness experience [1,2,3,5].

According to the World Health Organization, palliative care aims to improve the quality of life of patients and their families through comprehensive management of disease-related symptoms. It should be implemented at all stages of cancer, regardless of ongoing disease-modifying treatment, and adapted to patients’ changing needs [6]. Achieving these goals requires a holistic approach and close collaboration within multidisciplinary teams, in which pharmacological treatment is integrated with non-pharmacological interventions.

In recent years, increasing attention has been given to non-pharmacological interventions in cancer care [7]. One such form of support is an art-based intervention, understood as the therapeutic use of creative processes and artistic expression to promote patients’ well-being [8].

Previous studies have attempted to evaluate the efficacy of art-based interventions in palliative care. For example, a systematic integrative review examined the active engagement of terminally ill patients in artist-facilitated creative activities, as opposed to passive exposure to art [9]. However, the majority of research attention in this field has focused on music therapy. Existing systematic reviews and meta-analyses identified in our literature search have primarily assessed the effects of music therapy on pain, anxiety, depression, and quality of life [10,11,12,13].

Evidence regarding other forms of art-based interventions remains more limited. A systematic review conducted in pediatric palliative care reported psychophysical benefits associated with art therapy interventions [14]. Other systematic reviews have examined diverse art-based therapies, including drawing, painting, collage, clay modeling, autobiographical book creation, as well as active forms of music and dance therapy [15,16,17,18]. However, these reviews predominantly included oncology patients undergoing active treatment or cancer survivors, rather than adults receiving palliative care.

Importantly, authors of previous reviews consistently highlighted limitations such as the small number of available studies, substantial heterogeneity of art-based therapy interventions, variability in outcome measures, and the use of diverse assessment tools, all of which restricted the feasibility of quantitative synthesis [10,11,14,15,16,18].

To the best of our knowledge, despite growing interest in art-based interventions, no systematic reviews with meta-analyses have specifically focused on randomized controlled trials (RCTs) evaluating the efficacy of art-based therapy in adult patients with advanced cancer receiving palliative care. This lack of synthesized evidence limits the development of evidence-based clinical recommendations in palliative care settings.

Therefore, the present study aimed to conduct a systematic review and meta-analysis of RCTs assessing the effects of arts-based interventions, including music therapy, on selected aspects of functioning, symptom severity, and quality of life in adult patients with advanced cancer receiving palliative care.

## 2. Methods

### 2.1. Study Design

The systematic review and meta-analysis were conducted in accordance with the PRISMA (Preferred Reporting Items for Systematic Reviews and Meta-Analyses) 2020 guidelines [19]. The study protocol was prospectively registered in the international PROSPERO database under registration number CRD42025636049 on 27 January 2025. All deviations from the registered protocol are reported and justified in Supplementary Table S1.

### 2.2. Eligibility Criteria

Inclusion and exclusion criteria were formulated according to the PICOS framework.

P (participants): Studies involving adult patients aged 18 years or older with advanced cancer receiving palliative care were included. Trials with predominantly advanced-cancer populations were eligible if ≥95% of participants met the cancer criterion. Studies involving individuals under 18 years of age, patients not receiving palliative care, populations in which >5% of patients had non-oncological diseases, oncology patients undergoing curative treatment, cancer survivors, and studies involving caregivers, family members, or healthcare staff were excluded.

I (interventions): Studies using art-based interventions were included, such as music-based interventions, bibliotherapy, visual art therapy, drama therapy, poetry therapy, painting therapy, sculpting therapy, occupational therapy using art therapy, narrative art therapy, creative art therapy, expressive art therapy, dance therapy, dance-movement therapy, and movement-based art therapy. Studies were excluded if they used the following interventions: acupuncture, massage, hypnosis, aromatherapy, naturopathy, osteopathy, yoga, tai chi, marijuana treatment, curative oncological treatment (chemotherapy, radiotherapy, systemic therapy, hormone therapy), mind–body medicine, botanical medicine, nutritional therapy, manipulative practices, energy medicine, therapeutic touch, transcendental meditation, mindfulness-based medicine, mindful touch therapy, psychotherapy, virtual reality therapy, mindfulness therapy, Walkabout, Reiki, supportive-expressive therapy (Managing Cancer and Living Meaningfully), spiritual interventions, health promotion, traditional Chinese medicine, physiotherapy, collage therapy, reminiscence thinking, and narrative reminiscence therapy.

C (comparators): Studies were included if the control groups received only standard palliative care (including all conventional medical interventions for symptom management, pain relief, and supportive care) or an alternative intervention (such as relaxation and mindfulness techniques or neutral auditory interventions).

O (outcomes): Pain, fatigue, and sleep quality (measured using tools such as the Visual Analogue Scale (VAS), Fatigue Assessment Scale, and/or Pittsburgh Sleep Quality Index) are assessed. Anxiety and depression (measured using tools such as State–Trait Anxiety Inventory (STAI), Beck Depression Inventory, Profile of Mood States, and/or Hospital Anxiety and Depression Scale). Quality of life (measured using instruments such as the Hospice Quality of Life Index-Revised (HQOLI-R)).

S (study selection): RCTs were included. Exclusion criteria comprised intervention studies without randomization and/or control groups, non-interventional studies (observational, retrospective, or reviews), case studies, secondary analyses, and study protocols. Only studies published in English were included.

The application of the ≥95% threshold represents a deviation from the eligibility criteria specified in the registered protocol (Supplementary Table S1). This pragmatic threshold was introduced during the eligibility assessment to operationalize the concept of a predominantly cancer population while avoiding the exclusion of otherwise relevant trials due to a small proportion of participants without a cancer diagnosis.

### 2.3. Research Question

Based on the eligibility criteria, the research question was defined using the PICOS framework: In adult patients with advanced cancer receiving palliative care, do art-based interventions (such as music-based interventions, bibliotherapy, visual art therapy, drama therapy, poetry therapy, painting therapy, sculpting therapy, narrative art therapy, dance therapy, and movement-based art therapy), compared with no intervention or alternative interventions, affect outcomes such as quality of life, pain, fatigue, anxiety, and depression in RCTs?

### 2.4. Information Sources

A systematic literature search was conducted in PubMed, MEDLINE (Ovid), and EMBASE from database inception to 17 December 2024 (PubMed), 21 December 2024 (MEDLINE Ovid), and 10 January 2025 (EMBASE).

These initial searches were conducted before protocol registration and did not represent the final search used for study selection. The review protocol was prospectively registered with PROSPERO on 27 January 2025. At the time of registration, screening of records for eligibility, data extraction, risk-of-bias assessment, and data synthesis had not been initiated. An updated search of all databases was subsequently conducted to ensure that the evidence base was current. PubMed was re-searched on 20 February 2026, Ovid MEDLINE on 17 February 2026, and Embase on 24 February 2026 using the same search strategies and eligibility criteria as in the initial search. Reference lists of included publications were also screened to identify additional relevant studies. Trial registries and related sources, including ClinicalTrials.gov, the WHO International Clinical Trials Registry Platform (ICTRP), and the Cochrane Central Register of Controlled Trials (CENTRAL), were systematically searched on 12 August 2026, 13 August 2026, and 13 August 2026, respectively, to identify ongoing or unpublished studies. Google Scholar was searched on 16 August 2026 using 12 separate search strategies covering the predefined categories of art-based interventions. For each Google Scholar search strategy, when more than 300 results were retrieved, the first 300 results ranked by relevance were screened; when 300 or fewer results were retrieved, all retrieved results were screened. Full search strategies for all databases, including applied filters and limits, are presented in Supplementary Tables S2 and S3.

### 2.5. Search Strategy

The search included keywords such as the following:

((complementary therapy) OR (alternative therapy) OR (bibliotherapy) OR (narrative therapy) OR (narrative thinking) OR (poetry therapy) OR (poetry) OR (music therapy) OR (creative therapy) OR (painting therapy) OR (painting) OR (sculpting therapy) OR (sculpting) OR (art therapy) OR (healing art) OR (occupational therapy) OR (choreotherapy) OR (expressive therapy) OR (drama therapy) OR (dance therapy) OR (dance) OR (dance-movement therapy) OR (movement therapy) OR (play therapy) OR (creative art therapy) OR (visual art therapy) OR (movement psychotherapy)) AND ((cancer) OR (tumor) OR (neoplasms) OR (terminal cancer) OR (metastatic cancer) OR (malignant) OR (incurable disease)) AND ((palliative care) OR (palliative medicine) OR (hospice care) OR (terminal care) OR (end-of-life care) OR (hospice)).

The complete PubMed search strategy was as follows:

((complementary therapy[Title/Abstract]) OR (alternative therapy[Title/Abstract]) OR (bibliotherapy[Title/Abstract]) OR (narrative therapy[Title/Abstract]) OR (reminiscence bibliotherapy[Title/Abstract]) OR (narrative thinking[Title/Abstract]) OR (poetry therapy[Title/Abstract]) OR (music therapy[Title/Abstract]) OR (creative therapy[Title/Abstract]) OR (painting therapy[Title/Abstract]) OR (sculpting therapy[Title/Abstract]) OR (art therapy[Title/Abstract]) OR (healing art[Title/Abstract]) OR (occupational therapy[Title/Abstract]) OR (choreotherapy[Title/Abstract]) OR (expressive therapy[Title/Abstract]) OR (drama therapy[Title/Abstract]) OR (dance therapy[Title/Abstract]) OR (dance[Title/Abstract]) OR (dance-movement therapy[Title/Abstract]) OR (movement therapy[Title/Abstract]) OR (play therapy[Title/Abstract]) OR (creative art therapy[Title/Abstract]) OR (visual art therapy[Title/Abstract]) OR (narrative reminiscence therapy[Title/Abstract]) OR (movement psychotherapy[Title/Abstract])) AND ((cancer[Title/Abstract]) OR (tumor[Title/Abstract]) OR (neoplasms[Title/Abstract]) OR (terminal cancer[Title/Abstract]) OR (metastatic cancer[Title/Abstract]) OR (malignant[Title/Abstract]) OR (incurable disease[Title/Abstract])) AND ((palliative care[Title/Abstract]) OR (palliative medicine[Title/Abstract]) OR (hospice care[Title/Abstract]) OR (terminal care[Title/Abstract]) OR (end-of-life care[Title/Abstract]) OR (end of life care[Title/Abstract]) OR (hospice[Title/Abstract])). Filter: English.

The remaining search strategies for Ovid MEDLINE and Embase are available in the Supplementary Material (Supplementary Table S2), whereas the additional search strategies for Google Scholar, ClinicalTrials.gov, WHO ICTRP, and Cochrane CENTRAL are presented in Supplementary Table S3.

### 2.6. Selection of Records and Data Collection Processes

The inclusion and exclusion criteria were jointly developed by two reviewers (A.Ś. and A.O.-B.). All identified records were imported into EndNote, where duplicates were identified and removed (A.Ś.). The research selection process took place in two stages. In the first stage, titles and abstracts were screened for eligibility to identify articles for further assessment. In the second stage, the full texts of articles that passed the initial screening were evaluated.

The entire selection process was performed manually. Two reviewers independently assessed publications for inclusion in the systematic review based on the eligibility criteria at both stages (title/abstract and full-text level; A.Ś./J.N. and A.O.-B./M.G.). The reviewers were blinded to each other’s decisions. Any disagreements were resolved with a third reviewer (M.S. or A.P.) through discussion and consensus.

### 2.7. Data Items

Data extraction was performed from the full texts of the included studies. Two reviewers (A.Ś. and A.O.-B.) independently extracted the relevant data and entered them into standardized extraction forms. The extracted information was synthesized by the reviewers. The reviewers compared the extracted texts and any discrepancies were resolved through discussion until consensus was reached. The extracted variables included study characteristics (first author, year of publication, country, title, study design and randomization method), participants’ characteristics (sample size, age, sex, cancer diagnosis and care setting) (Table 1), characteristics of the intervention and control conditions (type of art-based intervention, number and duration of sessions), as well as outcome domains and measurement instruments (Table 2).

### 2.8. Risk of Bias Assessment and Study Quality

The risk of bias of the included randomized controlled trials was assessed using the Cochrane Risk of Bias 2 (RoB 2) tool [27]. This tool evaluates bias across five domains: the randomization process, deviations from intended interventions, missing outcome data, measurement of outcomes, and selection of the reported results. Each study was classified within these domains as having a ‘low’, ‘high’, or ‘some concerns’ risk of bias. In addition to the overall assessment of each included study, risk of bias was assessed at the outcome-result level for each outcome included in the meta-analysis and, where applicable, at the relevant time point.

Additionally, the methodological quality of the included randomized controlled trials was assessed using the Physiotherapy Evidence Database (PEDro) scale. The PEDro scale, which is scored from 1 to 10, is a validated tool for assessing methodological quality in RCTs [28]. Based on PEDro scores, studies were classified as having high (≥7 points), moderate (4–6 points), or low (≤3 points) methodological quality. Detailed PEDro scores for each study are presented in Supplementary Table S7.

The assessment was conducted independently by two reviewers (A.Ś. and M.S.), and disagreements were resolved through discussion, with the involvement of a third (A.P.) reviewer when necessary.

Funnel plots and Egger’s regression test were planned for assessing publication bias when at least 10 studies were available per outcome. However, publication bias was not assessed because the number of included studies in each meta-analysis was too small to allow a reliable evaluation.

We applied the Grading of Recommendations Assessment, Development and Evaluation (GRADE) approach to assess the certainty of evidence for the main outcomes included in the meta-analysis (pain intensity, quality of life). The quality of evidence was evaluated across five domains: risk of bias, inconsistency, indirectness, imprecision, and publication bias. The certainty of evidence was categorized as high, moderate, low, or very low. The assessment was performed by one reviewer (M.S.) and independently verified by a second reviewer (A.Ś.). Any disagreements were resolved through discussion and consensus. A Summary of Findings table was generated using the GRADEpro Guideline Development Tool (McMaster University, 2015; developed by Evidence Prime, Inc., Kraków, Poland; available from https://www.gradepro.org/) and is presented in Supplementary Table S4.

### 2.9. Synthesis Methods

Randomized controlled trials were eligible for inclusion in the meta-analysis if participants in the music-based interventions group received at least two therapeutic sessions. The outcomes included in the quantitative synthesis were pain intensity and quality of life measures.

For each outcome, post-intervention mean values and corresponding standard deviations (SDs) from the first assessment conducted after completion of the final music-based intervention session were extracted. When multiple post-intervention assessments were reported, the earliest available assessment following the final session was selected. Intention-to-treat data were used when available; otherwise, data from participants with available post-intervention assessments was used. The exact assessment time point used for each study and outcome is reported in Supplementary Table S5.

For multi-arm trials, only the intervention arm that most directly corresponded to the primary music-based intervention evaluated in the original study was included, along with the relevant control group, to avoid double-counting participants. For crossover trials, paired data were used when sufficient information was available to account for the within-participant design. If the mean values and corresponding SDs required for the meta-analysis were not reported, the study was excluded from the quantitative synthesis for that specific outcome.

For continuous outcomes, standardized mean differences (SMDs), expressed as Cohen’s *d* with 95% confidence intervals (CIs), were calculated when different measurement scales or non-comparable score metrics were used across studies to assess the same outcome. For quality of life, although both studies used the HQOLI-R, the reported scores were not directly comparable in their numerical metrics, and the available information did not allow equivalence of the raw score units to be established; therefore, SMDs were used rather than raw mean differences (MDs). No small-sample correction was applied.

A random-effects model was used because of anticipated clinical and methodological heterogeneity arising from differences in study populations, interventions, outcome measures, and trial settings. Random-effects meta-analyses were performed using the DerSimonian–Laird estimator for the between-study variance (τ^2^). Statistical heterogeneity was quantified using τ^2^ and I^2^ statistics. I^2^ values of approximately 25%, 50%, and 75% were interpreted as indicating low, moderate, and high heterogeneity, respectively.

Sensitivity analyses were performed by excluding studies judged to be at high risk of bias based on outcome-specific RoB 2 assessments to evaluate the robustness of the pooled effect estimates. When a mixed-population study contributed to a pooled outcome and cancer-specific data could not be extracted separately, we performed a sensitivity analysis excluding that study. Where sufficient data were available, subgroup analyses were planned according to (1) type of control group (music-based interventions vs. standard control; music-based interventions vs. active control) and (2) number of music-based intervention sessions. Given the very small number of studies within individual subgroups, analyses based on only one or two studies were considered exploratory and were interpreted descriptively. No conclusions regarding differences in treatment effects between subgroups were drawn.

Statistical significance was defined as *p* < 0.05. All analyses were conducted using Statistica software (Data Analysis Software System, version 13.3, Plus Set package; Statistica AWF Katowice, serial number JPZ009K288211FAACD-Q).

## 3. Results

### 3.1. Study Selection

**Figure 1:**
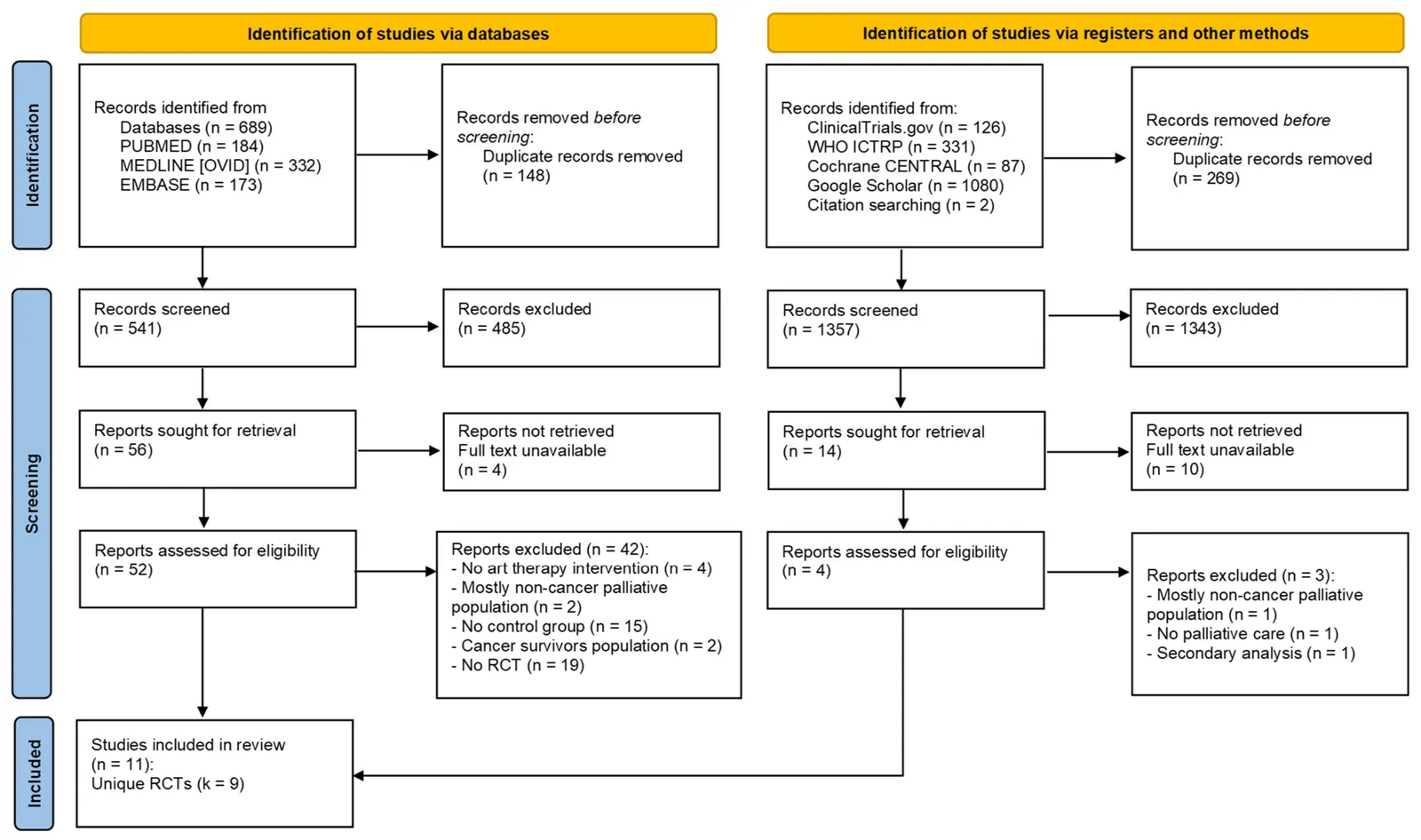
PRISMA flow diagram illustrating the study selection process.

A total of 689 records were identified through database searches (PubMed, MEDLINE Ovid, EMBASE). A total of 1626 records were identified through registers and other methods (ClinicalTrials.gov, WHO ICTRP, Cochrane CENTRAL, Google Scholar, and citation searching). In total, 2315 records were identified across all sources. Ultimately, 11 studies representing 9 unique RCTs meeting all eligibility criteria were included in the systematic review. The sources of evidence, numbers of records identified, duplicates removed, reports unavailable for retrieval, and full-text reports excluded, together with the reasons for exclusion, are presented in the PRISMA flow diagram (Figure 1). The counts presented in the PRISMA flow diagram for PubMed, MEDLINE (Ovid), and Embase correspond to the full rerun of the searches conducted in February 2026.

### 3.2. Study Characteristics and Quality Assessment

A total of 9 unique randomized controlled trials involving 675 randomized participants were included in the review. The characteristics of the included studies are summarized in Table 1. Details of the interventions, outcome measures, and main findings of the included studies are summarized in Table 2.

Of the 9 unique RCTs reported in 11 publications, the majority were conducted in hospital-based palliative care settings (k = 6 unique RCTs, reported in 8 publications) [3,5,20,21,22,23,24,26], while one RCT was conducted in a hospice setting [2], one in home hospice care [1] and one in home-based palliative care [28]. The RCTs originated from various countries, most commonly Germany (k = 2, reported in 4 publications) [3,5,24,27] and Spain (k = 2) [26,28], as well as Turkey (k = 1) [22], China (k = 1) [25], the United States (k = 1) [1], Australia (k = 1) [2], and Sri Lanka (k = 1) [23].

The majority of the RCTs were two-arm parallel-group trials (k = 7, reported in 9 publications) [1,2,3,5,20,22,24,25,26]; one RCT employed a three-arm design [23], and one used a crossover design [21].

Regarding patient populations, all participants were adults, and six RCTs included exclusively patients with cancer [1,20,21,23,24,25], whereas three RCTs reported in five publications involved predominantly cancer populations with a small proportion (<5%) of participants with non-cancer diagnoses [2,3,5,22,26].

All included studies evaluated music-based interventions. Within this overarching category, three types of interventions were distinguished. Music therapy was defined as interventions delivered by a qualified music therapist (k = 5 unique RCTs reported in 7 publications) [1,2,3,5,22,24,26]. A music-based intervention facilitated by a music specialist, whose qualifications as a music therapist were not reported, was identified in one unique RCT [20]. Music listening interventions were identified in three unique RCTs [21,23,25]. Independent prerecorded music listening was explicitly reported in one RCT [25], whereas in two RCTs the authors did not specify whether music listening was undertaken independently or in the presence of another person [21,23].

The music-based interventions were further categorized into passive and active or mixed approaches. Passive interventions, in which patients were not actively engaged musically and the primary mode of intervention was music listening, were applied in five RCTs and reported in six publications [3,20,21,23,25,26]. In contrast, four RCTs, reported in five publications [1,2,5,22,24], employed active or mixed approaches, in which patients not only listened to music but also engaged actively (e.g., through singing, interaction with the therapist, or reflective processing).

In six RCTs, reported in eight publications [1,2,3,5,20,22,24,26], music was delivered live, at least as one component of the intervention, whereas three RCTs [21,23,25] used prerecorded music.

The number of music-based intervention sessions varied across the RCTs. In three RCTs, a single intervention session was applied [2,21,24], while two RCTs reported in four publications involved two to three sessions [3,5,22,26]. Three RCTs involved more than three sessions [20,23,25]. In one RCT, the number of sessions varied individually, ranging from 2 to 13 sessions [1].

Both standard control groups, which involved no additional intervention beyond routine palliative care (k = 3 RCTs) [1,20,23], and active control groups, in which alternative interventions such as relaxation, mindfulness techniques, health education, conversation, or other forms of support were used (k = 7 RCTs, reported in 9 publications) [2,3,5,21,22,23,24,25,26], were applied. In one RCT, both a standard and an active control group were included [23].

**Figure 2:**
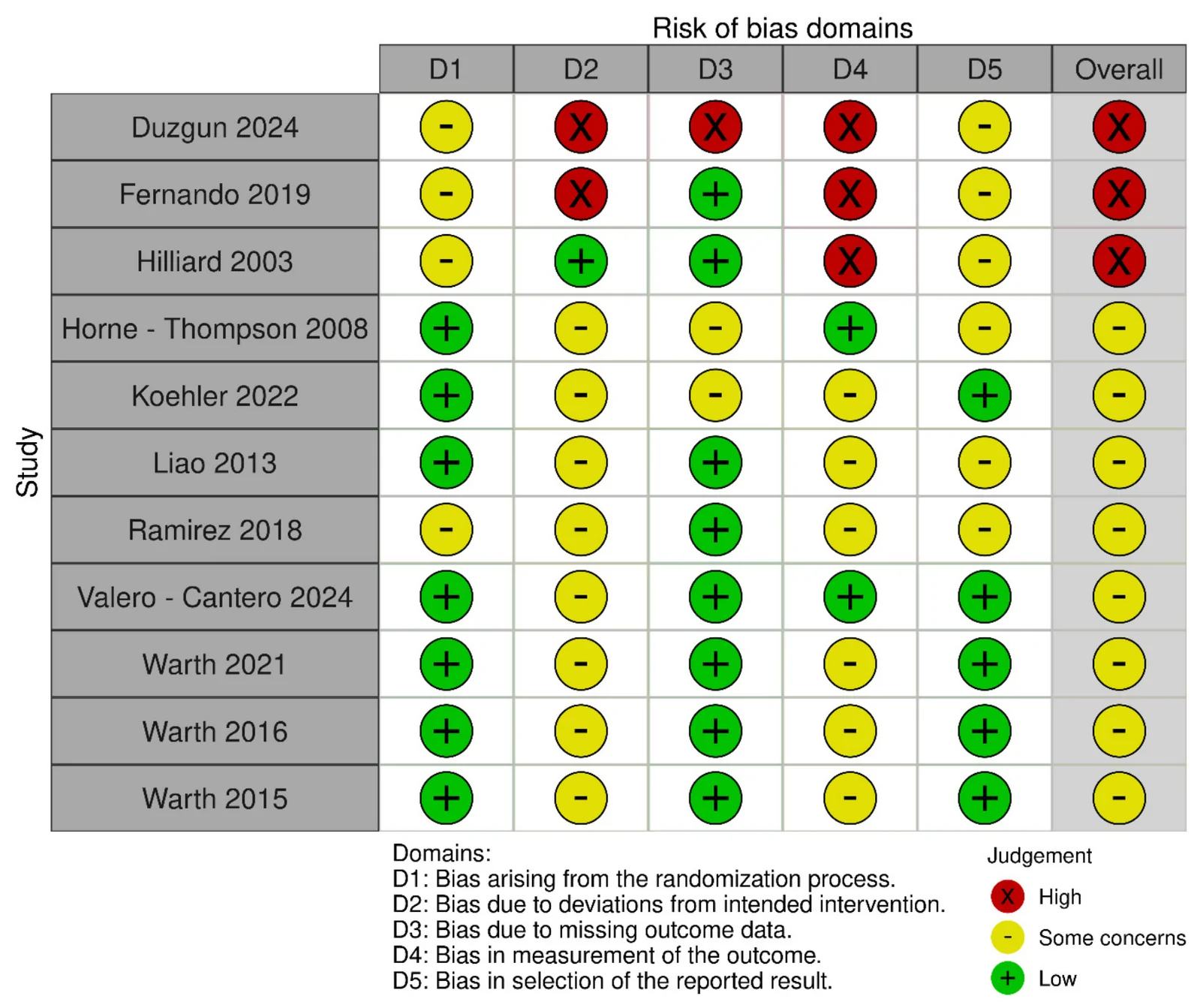

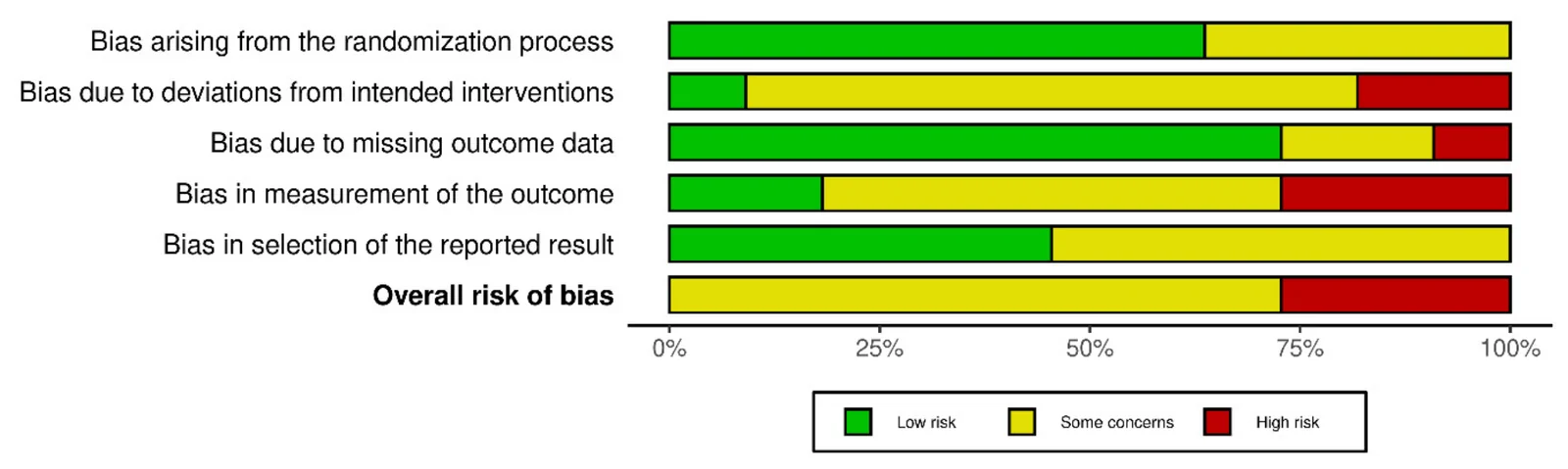
Risk of bias assessment of individual elements from five domains (D1–D5) across the 11 included publications [1,2,3,5,20,21,22,23,24,25,26].

None of the included publications were assessed as having a low risk of bias. Three reports [1,20,21] were judged to have a high risk of bias, while the remaining eight [2,3,5,22,23,24,25,26] raised some concerns regarding potential bias. The risk of bias across the included publications is summarized in Figure 2. Risk-of-bias assessments for specific outcomes and assessment time points of studies included in the meta-analyses are presented in Supplementary Table S6.

The methodological quality of the 11 included reports from 9 unique randomized controlled trials was assessed using the PEDro scale. Based on PEDro scores, six reports [2,3,5,22,23,25] were classified as having high methodological quality, five [1,20,21,24,26] as moderate quality, and none met the criteria for low methodological quality. Detailed PEDro scores are presented in Supplementary Table S7.

## 4. Results of Meta-Analyses

### 4.1. Pain Intensity

**Figure 3:**
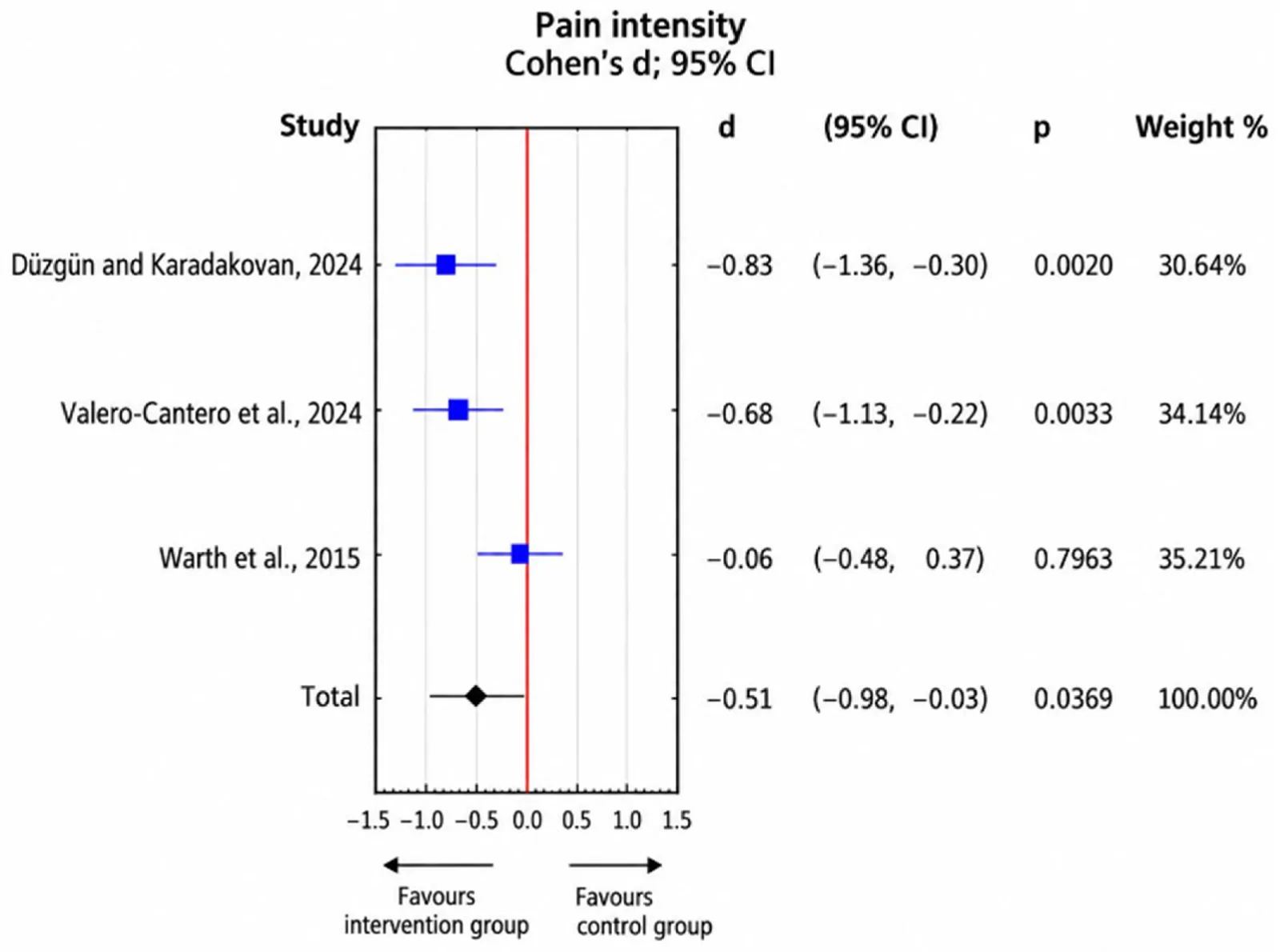
Forest plot comparing pain intensity between music-based interventions and control groups [3,20,25].

**Figure 4:**
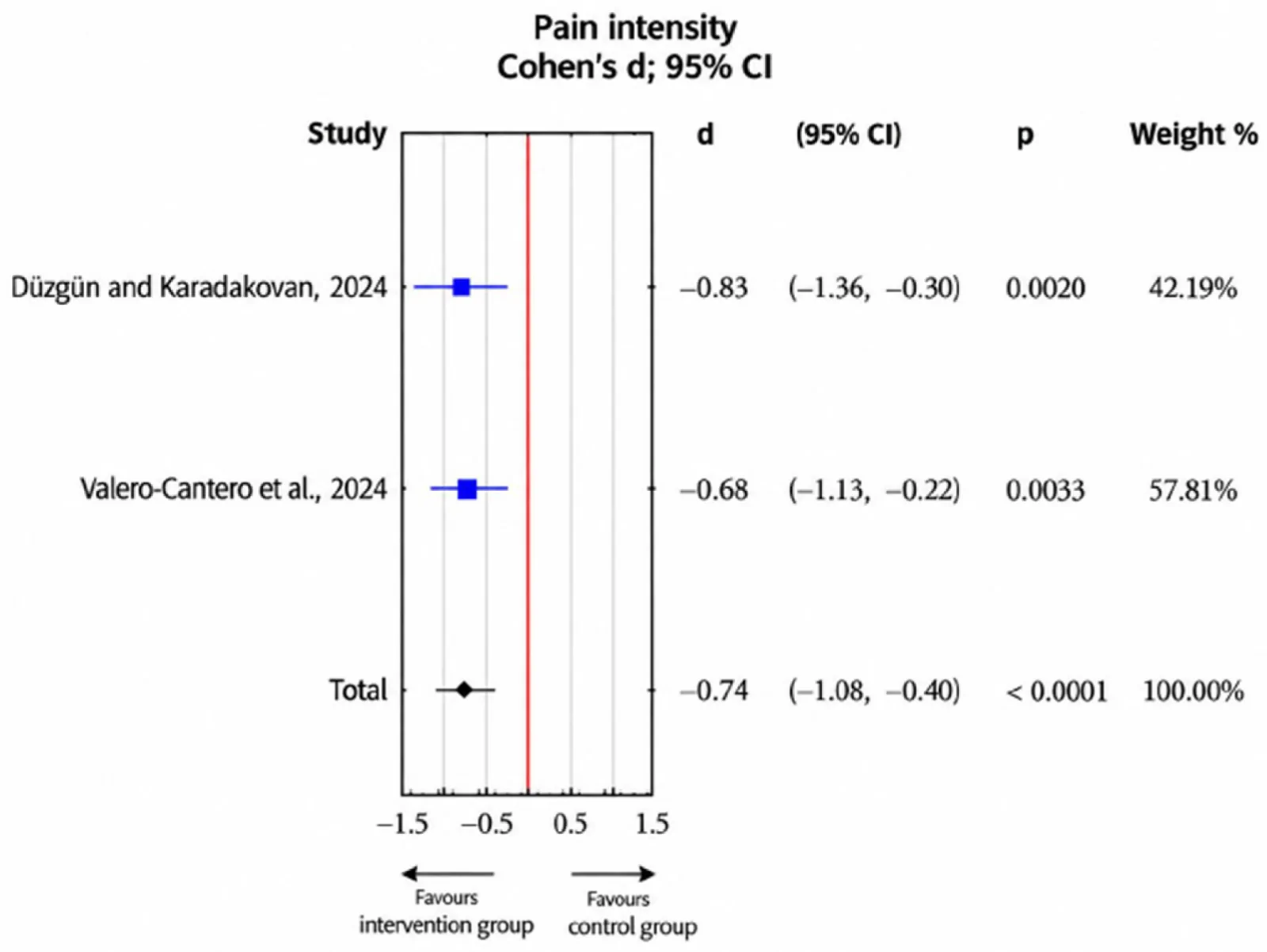
Forest plot of the sensitivity analysis for pain intensity after exclusion of the mixed-population study [20,25].

Three RCTs were included in the meta-analysis of pain intensity (*n* = 224 participants). The random-effects model showed a statistically significant reduction in pain intensity in favor of music-based interventions (SMD = −0.51; 95% CI: −0.98 to −0.03; *p* = 0.04) (Figure 3), with high between-study heterogeneity (I^2^ = 67.69%, τ^2^ = 0.119; *p* = 0.045). A sensitivity analysis excluding the study by Warth et al. [3], which included two participants without cancer and did not provide cancer-specific outcome data, yielded a larger pooled effect in favor of music-based interventions (SMD = −0.74; 95%CI: −1.08 to −0.40; *p* < 0.0001) (Figure 4), while heterogeneity decreased to 0% (I^2^ = 0%, τ^2^ = 0; *p* = 0.659; k = 2; *n* = 140 participants). In a separate sensitivity analysis excluding the study by Düzgün and Karadakovan [20], which was judged to be at high risk of bias for this outcome, the pooled effect was attenuated and no longer statistically significant (SMD = −0.36; 95%CI: −0.97 to 0.24; *p* = 0.24) (Supplementary Figure S1), with high heterogeneity remaining (I^2^ = 73.75%, τ^2^ = 0.141) (k = 2, *n* = 164 participants). According to the GRADE assessment, the certainty of evidence for pain intensity was very low (Supplementary Table S4).

Given the limited number of RCTs, findings according to control condition were summarized descriptively rather than being interpreted as a formal subgroup comparison. In the single study (*n* = 60 participants) using a standard control condition, music-based intervention was associated with lower pain intensity (SMD = −0.83; 95%CI: −1.36 to −0.30; *p* = 0.002). Across the two studies (*n* = 164 participants) using active control conditions, the pooled effect estimate was SMD = −0.36 (95%CI: −0.97 to 0.24; *p* = 0.24) (Supplementary Figure S2).

In an exploratory descriptive analysis according to the number of music-based intervention sessions, the pooled effect estimate from the two studies (*n* = 140 participants) using >3 sessions was SMD = −0.74 (95% CI: −1.08 to −0.40; *p* < 0.001), whereas the single study (*n* = 84 participants) using ≤3 sessions showed an effect estimate of SMD = −0.06 (95% CI: −0.48 to 0.37; *p* = 0.796) (Supplementary Figure S3). Given the very small number of studies, these findings should not be interpreted as evidence of differences in treatment effects between subgroups.

### 4.2. Quality of Life (HQOLI-R)

**Figure 5:**
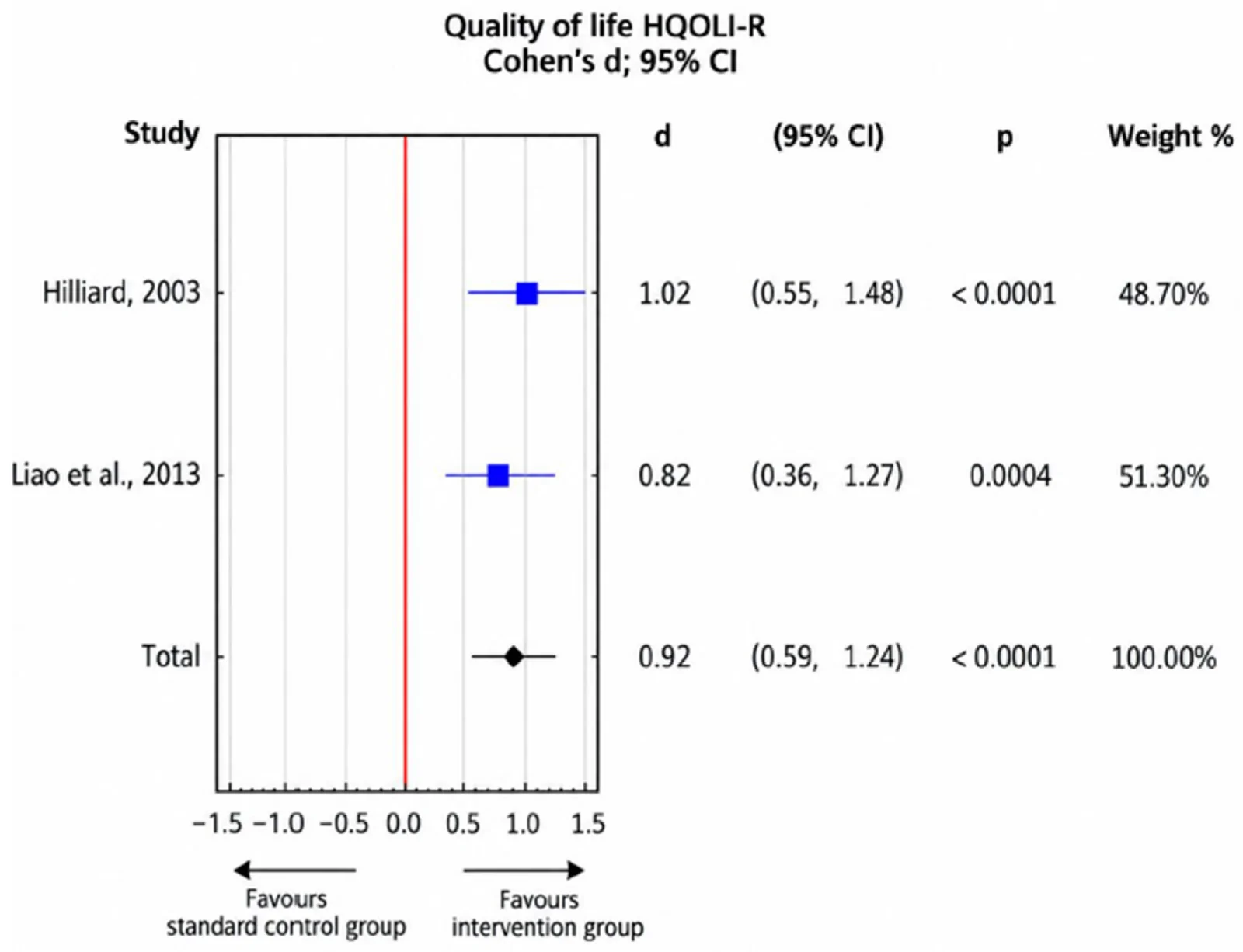
Forest plot comparing quality of life, as assessed by the HQOLI-R, between the music-based intervention and standard control groups [1,23].

For quality of life assessed using HQOLI-R, music-based interventions were associated with significantly higher quality-of-life scores compared with the standard control condition (SMD = 0.92; 95% CI: 0.59 to 1.24; *p* < 0.0001) (Figure 5), with no observed heterogeneity (I^2^ = 0%, τ^2^ = 0; *p* = 0.55) (k = 2; *n* = 168 participants). However, the quality-of-life result in Hilliard [1] was judged to be at high risk of bias. According to the GRADE assessment (Supplementary Table S4), the certainty of evidence was very low; therefore, the evidence for an effect of music-based interventions on quality of life is highly uncertain.

## 5. Discussion

This systematic review and meta-analysis aimed to evaluate the efficacy of art-based interventions in adult patients with advanced cancer receiving palliative care. Despite a broad search strategy, all included RCTs investigated only music-based interventions, which indicates a clear gap in the literature regarding other forms of art-based interventions in this population.

The pooled analysis suggests a possible reduction in pain intensity among patients receiving music-based interventions as an addition to routine palliative care compared with control conditions; however, the certainty of this evidence is very low. Given that pain is one of the most common and burdensome symptoms among patients with advanced cancer, this finding may have potential clinical relevance; however, it should be interpreted with considerable caution. These findings are consistent with previous meta-analyses by Gao et al. [11] and McConnell et al. [10], which also reported a reduction in pain intensity associated with music-based interventions in end-of-life care.

However, it should be emphasized that the observed effect on pain intensity is sensitive to risk of bias. In a sensitivity analysis excluding one study [20], which was judged to be at high risk of bias for this outcome, the pooled effect was attenuated and was no longer statistically significant, with high heterogeneity remaining. This finding indicates limited robustness of the pooled effect and warrants cautious interpretation, particularly given the very low certainty of evidence according to the GRADE assessment.

For quality of life assessed using the HQOLI-R, music-based interventions were associated with significantly higher quality-of-life scores compared with the standard control condition. However, this finding should be interpreted with considerable caution, as it was based on only two studies, and the quality-of-life result in Hilliard [1] was judged to be at high risk of bias. Moreover, according to the GRADE assessment, the certainty of evidence was very low, indicating that the estimated effect on quality of life is highly uncertain. Therefore, the available evidence does not allow firm conclusions regarding the effect of music-based interventions on quality of life. These findings are broadly consistent with the meta-analysis by Gao et al. [11], which also reported significant improvements in quality of life in terminally ill patients. However, differences in eligibility criteria, including broader patient populations and the inclusion of both English- and Chinese-language studies in that review, should be considered when comparing the findings.

A major factor limiting the interpretation of the present findings is the high heterogeneity across studies. This variability likely resulted from both clinical and methodological differences. As indicated by the characteristics of the included reports, music-based interventions were delivered in highly diverse ways—from passive listening to music [3,20,21,23,25,26] to therapist-led sessions incorporating elements of relaxation, reminiscence, and active patient engagement in musical activities [1,2,5,22,24]. The interventions also differed in duration, number of sessions, and frequency, which may have influenced the observed therapeutic effects.

Additional potential sources of heterogeneity included cancer types, the use of live versus prerecorded music, differences in intervention settings—including hospital, hospice, and home-based palliative care—as well as differences in control conditions and outcome assessment instruments. However, the contribution of these characteristics to the observed heterogeneity could not be formally evaluated because of the limited number of studies available for quantitative synthesis.

Another important factor limiting the interpretation of the findings is the risk of bias. According to the RoB 2 assessment, none of the included reports were rated as having a low risk of bias, with three reports [1,20,21] classified as high risk and the remaining reports [2,3,5,22,23,24,25,26] raising some concerns. Outcome-specific RoB 2 assessments were also performed for the results included in the meta-analyses of pain intensity and quality of life. The pain result reported by Düzgün and Karadakovan [20] and the quality-of-life result reported by Hilliard [1] were judged to be at high risk of bias, whereas the remaining results included in the quantitative syntheses raised some concerns. This substantially reduces confidence in the observed effects. Although PEDro scores indicated moderate to high methodological quality across reports, these findings should be interpreted with caution, as methodological quality does not necessarily reflect the risk of bias. In addition, the small number of unique RCTs and the limited number of reports contributing to individual meta-analyses reduce the robustness and precision of the available evidence and preclude definitive conclusions.

Publication bias was not formally assessed in the present review because fewer than 10 studies were available for each meta-analysis, making funnel plot interpretation and Egger’s regression test unreliable. Consequently, the possibility of publication bias and small-study effects cannot be excluded. This limitation is particularly relevant because the non-publication or non-identification of studies reporting null or unfavorable results may have biased the pooled effect estimates. Therefore, the magnitude and direction of the pooled effects should be interpreted with caution, and future updates of this review should reassess publication bias when a sufficient number of studies become available.

In the present review, the included studies involved populations consisting predominantly of patients with advanced cancer receiving palliative care. In three of the nine unique RCTs, reported in five publications, a small proportion (<5%) of participants had non-cancer diagnoses [2,3,5,22,26].

Despite these limitations, the findings may have potential clinical relevance. Music-based interventions may represent a complementary non-pharmacological approach in palliative care; however, the very low certainty of evidence and limited robustness of the pooled pain effect preclude firm conclusions regarding clinical benefit. Further well-designed trials are needed to determine whether the observed effects translate into clinically meaningful improvements for patients with advanced cancer receiving palliative care.

An important observation arising from this review is the lack of randomized controlled trials evaluating other forms of arts-based interventions, including visual art therapy (e.g., painting, drawing, sculpture), dance therapy, poetry therapy, bibliotherapy, dramatherapy, and other expressive therapies. This indicates a clear research gap and suggests that the scientific evidence for other art-based interventions in palliative care for patients with advanced cancer remains very limited.

Future research should focus on well-designed, adequately powered randomized controlled trials with more standardized intervention protocols and measurement tools. Where feasible, longer follow-up periods should be included to better assess the durability of therapeutic effects. However, it should be recognized that the realities of palliative care—including limited life expectancy and rapid health deterioration—often make long-term follow-up difficult or impossible. Therefore, particular attention should also be given to clinically meaningful short- and mid-term outcomes, which may best reflect the needs of this patient population.

## 6. Limitations

This systematic review has several limitations that should be considered when interpreting the findings. First, although the literature search was expanded beyond the three main electronic databases to include additional trial registries and other sources, PsycINFO and CINAHL were not searched. Given the psychological, nursing, and arts-related nature of the topic, this may have resulted in the omission of potentially relevant studies indexed in these databases. Second, only studies published in English were included, which may have led to the exclusion of potentially relevant randomized controlled trials published in other languages. This language restriction may have introduced language bias and reduced the comprehensiveness and generalizability of the findings. The review was restricted to randomized controlled trials, which, although they are considered the gold standard for evaluating intervention efficacy, may have limited the inclusion of other relevant evidence and reduced the overall breadth of available data. Despite a broad search strategy covering multiple forms of art-based interventions, no eligible RCTs evaluating non-music art-based interventions were identified. Therefore, the conclusions are limited to music-based interventions and cannot be generalized to other art-based therapies in advanced cancer palliative care. In addition, exploratory descriptive analyses according to control condition and the number of music-based intervention sessions were based on only one or two studies per subgroup. Therefore, these analyses should not be interpreted as formal tests of subgroup differences, and no conclusions regarding differences in intervention effects between subgroups can be drawn.

## 7. Conclusions

Randomized evidence regarding art-based interventions in advanced cancer palliative care is limited to music-based interventions. However, the very low certainty of evidence, high heterogeneity, and limited robustness of the pooled findings preclude firm conclusions regarding clinical benefit. No firm conclusions can therefore be drawn regarding the effects of music-based interventions on pain intensity or quality of life.

An important conclusion arising from this review is the lack of randomized controlled trials evaluating forms of art-based therapies other than music-based interventions, which indicates a clear research gap. Further high-quality randomized controlled trials with adequate sample sizes, standardized intervention protocols, and consistent outcome measures are needed to better define the role of art-based interventions in palliative care for patients with advanced cancer.

## Figures and Tables

**Table 1:** Characteristics of the 11 included reports from nine unique randomized controlled trials of music-based interventions.

First Author, Year, Country, Trial Registration Number	RCT Type and Randomization Method	Participants’ Characteristics		
		Primary Diagnosis	Context	Number, Age, Gender, Group Division (IG vs. CGs/CGa)
Düzgün 2024 [20] Turkey NCT04486443	2-arm design; participants stratified by age, gender, and disease stage; IG determined by coin toss.	Advanced cancer (stage III: *n* = 15, stage IV: *n* = 45, 75%): lung, pancreas, gastric, colon, breast	Hospitalized palliative care patients; no terminal or bedridden status; pain ≥ 3 (VAS); on medications, narcotic analgesics before, during, after research.	Randomized: *n* = 78, analyzed: *n* = 60. IG: *n* = 30, CGs: *n* = 30; age: 40–80, females: *n* = 12, males: *n* = 48; age: 40–50 (IG: *n* = 5, CGs: *n* = 5); age: 51–60 (IG: *n* = 6, CGs: *n* = 6); age: 61–70 (IG: *n* = 14, CGs: *n* = 14); age: 71–80 (IG: *n* = 5, CGs: *n* = 5)
Fernando 2019 [21] Sri Lanka. SLCTR/2016/006.	Open, crossover design; sample size calculated; sample selected from the 39 eligible patients through computer-assisted simple randomization	Advanced cancer: hematological, respiratory tract, gastrointestinal, gynecological, endocrine, male reproductive, musculoskeletal	Hospitalized palliative care patients with basal levels of pain ≥ 1/10 (VAS) for >12 weeks	Randomized/analyzed: *n* = 24. The same group was initially subjected to “control” and subsequently to “intervention” phase; age: 38–80 (females: *n* = 12, males: *n* = 12)
Hilliard 2003 [1] The USA Number not found.	2-arm design; gender and age controlled, with equal numbers of males/females and patients ≥65 and <65 (since older patients typically report less pain).	Terminal cancer of: lung, colon, kidney, nasopharynx, prostate, liver, esophagus, breast, pancreas, brain, oral cavity, ovary, stomach, endometrium, sinus, larynx, rectum, adeno, leukemia, melanoma, unspecified cancer (prognosis ≤ 6 months of lifespan).	Patients receiving hospice care at home for terminally ill patients and their families (newly admitted patients)	Randomized/analyzed: *n* = 80. IG: *n* = 40 (mean age: 66), CGs: *n* = 40 (mean age: 65). Design controlled for gender and age in each group, (females: *n* = 20, males: *n* = 20; age: <65: *n* = 20, >65: *n* = 20).
Horne-Thompson 2008 [2] Australia Number not found.	2-arm design (numbered envelope system); randomization by a university statistical service. After the participant’s written consent, the envelope was opened to reveal group allocation. A pretest–posttest design. The music therapist involved in IG and volunteer assigned to CGa not blinded to study purpose. Measurements and data collected by an independent staff member not involved with the study (most commonly the primary nurse).	End-stage terminal cancer (*n* = 24, 96%): bowel, breast, glioblastoma, lung, lymphoma, mesothelioma, metastatic melanoma, non-Hodgkin’s lymphoma, non small cell lung, esophageal, ovarian, rectal, thyroid, amyloidosis; Non-cancer end-stage disease (*n* = 1): chronic cardiac failure.	Patients under palliative care by inpatient hospice services	Randomized/analyzed: *n* = 25; age:18–90, mean age: 73.9 ± 13.32; IG: *n* = 13 (mean age: 76.2 ± 10.36), CGa: *n* = 12 (mean age: 71.4 ± 16.05); males: *n* = 14 (IG: *n* = 8, CGa: *n* = 6), females: *n* = 11 (IG: *n* = 5, CGa: *n* = 6)
Koehler 2022 * [22] (Epub 2021). Germany DRKS00015308	2-arm multicenter parallel design; computer-generated block randomization (block size = 8), stratified by site; allocation concealed using sequentially numbered opaque envelopes opened after baseline assessment; participants blinded (CGa unaware of intervention); therapist/assessor blinding not feasible (single-blind).	Advanced cancer (*n* = 87, 97.8%): gastrointestinal, gynecologic, skin, lymphatic, thoracic, other; non-cancer (*n* = 2)	Hospitalized patients under specialized palliative treatment, or with an estimated survival < 12 months	Randomized: *n* = 104, analyzed: *n* = 89. IG: *n* = 44, CGa: *n* = 45; mean age: 65.8 (IG: 68.07 ± 11.52; CGa: 63.58 ± 11.98); females: IG: *n* = 34, CGa: *n* = 32.
Liao 2013 [23] China. NCT00964314.	Randomization sequence generated via statistical software (2:2:1 ratio) to assign patients to 3 groups; allocation concealed in sealed opaque envelopes opened upon inclusion. Single-blind design: subjects blinded, researchers aware of interventions.	Advanced cancer (based on tumor node metastasis, 7th edition of the American Joint Committee on Cancer, guidelines of 2010 European Society of Medical Oncologists).	Hospitalized patients, estimated survival ≤ 3 months, no psychiatric diseases, intellectual disability, deafness; KPS ≥ 60.	Randomized: *n* = 160. IG: *n* = 66, mean age: 63.09 ± 12.46; CGa: *n* = 63, mean age: 63.52 ± 14.65; CGs: *n* = 31, mean age: 62.68 ± 13.87 (females: *n* = 83, males: *n* = 77). Analyzed: *n* = 146. IG: *n* = 57, CGa: *n* = 58, CGs: *n* = 31
Ramirez 2018 [24] Spain Number not found.	2-arm design; patients assigned to IG or CGa via randomly permuted blocks.	Advanced cancer (type not specified)	Hospitalized palliative care patients with normal hearing	Randomized/analyzed: *n* = 40; mean age: 69 ± 15, females: *n* = 13, males: *n* = 27; IG: *n* = 20, CGa: *n* = 20
Valero-Cantero 2024 [25] Spain NCT04052074.	Double-blind multicenter trial; after enrollment, participants randomly assigned to IG or CGa using 40 sealed, opaque, numbered envelopes per group. Envelopes were shuffled, numbered, and opened sequentially by a researcher according to patient arrival. Both evaluators and patients were blinded to group allocation.	Advanced cancer: colon, lung, breast, pancreas, rectal, prostate, liver, oropharyngeal, kidney, lymphoma, bladder, brain, cervical, ovarian	Patients with noncurable disease under palliative care at home.	Randomized/analyzed: *n* = 80; mean age: 71.83 ± 7.77. IG *n* = 40 (mean age: 70.51 ± 7.16), CGa *n* = 40 (mean age: 73.14 ± 8.22); male: *n* = 44 (55.0%): IG: *n* = 25 (62.5%), CGa: *n* = 19 (47.5%); female: *n* = 36 (45.0%), IG: *n* = 15 (37.5%), CGa: *n* = 21 (52.5%).
Warth * Germany, 2021 [5] DRKS00015308	2-arm computer-generated block randomization (block size = 8), stratified by site, prepared by an independent researcher; allocation concealed using sequentially numbered opaque envelopes opened after baseline assessment; participants blinded (CGa unaware of allocation and hypothesis); baseline expectancy assessed (agreement 1–5); therapist/assessor blinding not feasible.	Advanced cancer (*n* = 102, 98.1%): gastrointestinal, gynecologic, skin, lymphatic, thoracic, other; non-cancer (*n* = 2)	Hospitalized patients at the palliative care unit + family members/close persons (recruited to assess treatment satisfaction).	Randomized/analyzed: *n* = 104 (ITT); mean age: 66.1 ± 12. IG: *n* = 52, mean age: 67.75 ± 11.5, CGa: *n* = 52, mean age: 64.46 ± 12.37; females: *n* = 77 (74%), IG *n* = 41, CGa *n* = 36. Completed: *n* = 81 (IG: *n* = 41, CGa: *n* = 40)
Warth ** Germany, 2016 [26] DRKS00006137	2-arm parallel design; computer-generated permuted block randomization with allocation concealed via numbered opaque envelopes; randomization and outcome assessment conducted by an assistant not involved in interventions.	Progressive, life-threatening disease advanced cancer (*n* = 82, 97.6%), cancer type not specified; non-cancer (*n* = 2)	Hospitalized patients under palliative care; no: final phase, cognitive, hearing impairments, signs of restlessness or agitation.	Randomized/analyzed: *n* = 84; mean age: 63 ± 13.4. IG: *n* = 42, mean age: 63.8 ± 14.1, CGa: *n* = 42, mean age: 62.2 ± 12.8; males: *n* = 24, females: *n* = 60 (IG *n* = 28; 66.7%, CGa: *n* = 32; 76.2%).
Warth ** 2015, Germany [3] DRKS00006137	2-arm design (IG vs. CGa), both interventions presented as equally effective; pre-generated computer block randomization (block size = 6); allocation concealed using sealed, numbered envelopes opened after baseline assessment, independently of treatment staff; participants blinded to hypotheses; therapist/assessor blinding not feasible.	Malignant tumor (*n* = 82, 97.6%), most common cancer: breast, pancreatic, ovarian, prostate; non-cancer (*n* = 2)	Hospitalized patients under palliative care according to OPS 8–892 or OPS 8–98e	Randomized: *n* = 84 (mean age: 63 ± 13.4, 71.4% females). IG: *n* = 42, CGa: *n* = 42. Analyzed: *n* = 78 (primary endpoint), *n* = 68 (psychometric outcomes), and *n* = 76 (physiological outcomes).

Abbreviations: CGa, active control group; CGs, standard control group; IG, intervention group; KPS, Karnofsky Performance Status; OPS, Operationen- und Prozedurenschlüssel (German procedure classification system); VAS, Visual Analogue Scale; ITT, intention-to-treat analysis. * Reports by Warth 2021 [5] and Koehler 2022 [22] are from the same RCT (DRKS00015308). ** Reports by Warth 2015 [3] and Warth 2016 [26] are from the same RCT (DRKS00006137).

**Table 2:** Intervention types, outcome measures, and main findings of the 11 included reports on music-based interventions.

First Author, Year, Country:	Intervention Characteristics				Outcomes Measures		Main Findings
	IG	CGs/CGa	Duration/Frequency	Timing of Assessments	Indicator	Instruments	
Düzgün 2024 [20] Turkey.	MT: Turkish classical music accompanied by a tambour (Hejaz or Rast modes according to patient’s preference) + Analgesic treatment and routine nursing care.	CGs: analgesic treatment and routine nursing care	IG: Six 10 min music sessions + analgesic treatment (on different days) CGs: Six analgesic treatment sessions (on different days). Intervention frequency was not reported.	Baseline: Patient Information Form, SF-MPQ. Baseline, 5, 30, 60 min after analgesic treatment at each session: Patient Follow-Up Form. Baseline, 3, 6 months after intervention: GCS, STAI, KPS	total pain, anxiety, comfort, functional capacity	Patient Information Form; SF-MPQ; Patient Follow-Up Form, GCS, STAI, KPS.	Turkish music + medicine was effective on pain, anxiety, comfort, and functional capacity. It could be achieved by nursing independently
Fernando 2019 [21] Sri Lanka.	Intervention phase (B), day 2: music intervention (listening): patients lay in bed (eyes blindfolded), listened to culturally appropriate instrumental classical music through earphones	Control phase (A), day 1: patients lay in bed (eyes blindfolded) and wore earplugs	One 30 min session: 08:00–08:30 am (music intervention: 28 min)	All parameters recorded at 08:00 am, 08:30 am and four hourly over 24 h in each phase	pain, anxiety, grief, PR, BP, RR, and pupillary size	VAS augmented with WBFPS: (pain, anxiety, grief); Palpation: radial PR; Mercury sphygmomanometry: systolic and diastolic brachial arterial BP; Visual measurement: RR; Visual observation: pupillary size	Music listening + ongoing therapies: Alleviated: pain, anxiety (4 h), low mood (12 h); reduced: pupillary size (12 h), respiratory rate (8 h)
Hilliard 2003 [1] USA	Clinical MT: live music (subject-preferred) + routine hospice services. MT: song choice, music-prompted reminiscence, singing, live music listening, lyric analysis, instrument playing, song parody, singing with accompaniment (iso principle). Funerals, memorial services planning, song gifts, music-assisted supportive counseling	CGs: routine hospice services	At least 2 sessions (2–13 sessions: subjects died at varying intervals); counselor determined visits frequency, weekly or bimonthly, This is based on a psychosocial assessment. Frequency of MT visits Same as counselor visits.	HQOLI-R and PPS: every visit. At least two QOL and physical status assessments	QOL; functional status; length of life; Relationship to time of death from the last music therapist or family support counselor visit.	HQOLI-R; PPS; length of life from hospice admission to death; period from last scheduled music therapist/counselor visit to death (days)	IG: higher QOL scores after S1, further increase after S2. CGs: lower scores after counselor visit 2 (IG: PPS scores declined after S3, QOL stable). IG: QOL higher even with physical function decline: MT improved QOL upon hospice admission and remaining lifetime. No role of age in QOL. Physical decline not affected by age or gender
Horne-Thompson 2008 [2] Australia.	MT: methods chosen by registered music therapist in consultation with the patient. Techniques: live familiar music, singing, music and relaxation/imagery, improvisation, music-assisted counseling, reminiscence, recorded music listening	CGa: routine activities with a volunteer (reading, conversation, emotional support; no music). Patients continued MT services outside of the study	A single 20–40 min MT session (adjusted for the clinical state of the patient)	ESAS: immediately before and after the intervention. HR: pre- and post-measurements	Anxiety, pain, tiredness, drowsiness, nausea, depression, appetite, well-being, and shortness of breath. HR	ESAS; Pulse oximeter HR	IG: single session MT reduced anxiety, pain, tiredness, and drowsiness: MT can be effective in managing tiredness and drowsiness. A single MT session can improve QOL. No change in HR was observed in the IG and CGa.
Koehler 2022 [22] Germany.	SOL + usual care; S1: choosing biographically meaningful, emotionally arousing SOL. S2: listening to live lullaby style SOL-guitar or e-piano (recorded). S3: receiving recording; discussing feelings and memories by pre-defined questions	CGa: relaxation/mindfulness therapy + usual care; S1: muscle relaxation. S2: mindful breathing, S3: imagery. End of session: debriefing on arising feelings, thoughts	Three 20–30 min sessions	NCCN DT (modified)—before, after each session. sCort and sAA–S2: Three samples in 20 min intervals, before, after session, at follow-up (between 2 and 6 pm, repeated to minimize variance due to diurnal cortisol patterns). Continuous PPG–S2 and 20 min later (follow-up 5 min segment)	Momentary distress; sCort, sAA; cardiac autonomic responses and autonomic activity	NCCN DT (modified): distress; Salivette^®^: saliva sampling; ELISA: sCort; kinetic colorimetric assay: sAA; PPG: cardiac autonomic response—beat-to-beat variations in HR, derived IBI between successive heartbeats in ms for 3 time segments of 5 min duration; RMSSD: parasympathetic activity, ability to recover; vagally mediated HRV, mHR: autonomic activity	SOL group: greater reduction in momentary distress compared to mindfulness exercise group. No differential treatment effects on sCort, sAA, mean HR, and HRV
Liao 2013 [23] China	Listening to Chinese medicine 5-element MT: elements: wood, fire, earth, metal, water; tunes: Jue, Zhi, Gong, Shang, Yu	CGa: Listening to Western music from the album “The Best of Chris Rea”. CGs: No music listening.	30 min session, 5 days a week for 3 weeks	HQOLI-R, KPS: before first treatment, at 1, 2, 3, and 4 weeks after first treatment. Symptom diary: before the first treatment, and then every 3 days starting after the first treatment	Quality of Life; Functional status.	HQOLI-R; KPS; Symptom diary score: functional status in advanced cancer patients	Chinese medicine 5-element MT: Improved QOL and KPS, in seniors and non-seniors and subjective symptoms in non-seniors
Ramirez 2018 [24] Spain.	MT: receptive and active songs, relaxation/imaginative receptive method; 3 music therapists	CGa: Conversation on music and music preferences (with 3 music therapists involved in MT for IG)	One 30 min session	EEG of 5 conditions: initial–before; passive listening, active listening, relaxation–during; final–after intervention. ESAS: pre- and post-intervention	brain activity; emotional well-being state	EEG; ESAS	IG: post-intervention, increased EEG valence, arousal values (positive MT emotional effect) and well-being; reduced tiredness, anxiety, breathing difficulties
Valero-Cantero 2024 [25] Spain.	Music intervention + standard care; pre-recorded music via mobile phone/MP3; music bringing good emotions, memories, chosen by patient from playlist. No health professional during sessions. Selected music types: Flamenco (28%), Classical (22%), Latinpop (12%), Blues and Pop (8% each), Opera (6%), Soul, Romantic ballad, Disco, Rockandroll (4% each)	CGa: audio sessions + standard care; pre-recorded health education by palliative care nurse via mobile phone/MP3. No health professional during sessions	7 half-hour sessions on consecutive days; in the mornings, at break time	ESAS: before and immediately after the 7-day intervention; CSQ-8: immediately after the intervention	pain, fatigue, nausea, depression, anxiety, drowsiness, dyspnea, appetite, well-being, sleep; Patient satisfaction with the healthcare received.	ESAS; CSQ-8	Benefits of individually selected music intervention in home-based palliative care. IG (vs. CGa): improved total ESAS symptom score, higher healthcare satisfaction (CSQ-8); decreased pain, anxiety, and depression; and increased well-being and overall emotional score. reduced fatigue, improved appetite, sleepiness, sleep, and physical score. Individually selected music intervention has the potential to enhance home care
Warth 2021 [5] Germany	Biographical SOL+ usual care; S1: choosing biographically meaningful, emotionally arousing SOL. S2: listening to lullaby-style SOL-guitar or e-piano + voice (recorded). S3: listening to recording, reflecting on feelings, memories to pre-defined questions. Receiving recording	CGa: Relaxation therapy + usual care—3 sessions: muscle relaxation, breathing, imaginary journey, brief inquiry at the end of each session	Three 20–30 min sessions, on 3 consecutive days (some deviations for organizational reasons or patient’s will)	S1: Demographical data/medical record S1, S3: MQOL-R subscale, BMGE, FACIT-Sp. S1, S2, S3: NCCN DT (pre- to post-session). S3: Feedback Questionnaire: patients and family (8–16 weeks after—family)	quality of life, acceptance and sense of meaning regarding one’s past life, non-religious aspects of spiritual well-being, momentary distress, Perception of treatment satisfaction.	MQOL-R: psychological subscale; BMGE: ego-integrity subscale; FACIT-Sp: 8-item meaning/peace-scale; NCCN DT (modified): momentary distress; Feedback Questionnaire (modified): patient and family member version; 8 items (1–5 agreement) on perception of treatment satisfaction	IG (SOL group): higher spiritual well-being and ego-integrity; There were no differences regarding quality of life compared to CGa.
Warth 2016 [26] Germany	Live MT (receptive); listening to improvised monochord play (supine position)- first 15 min [body scan exercise (3 min), vocal improvisation (12 min)], feedback conversation on experience (5 min)	CGa: body scan, meditation via headphones (supine position)— pre-recorded mindfulness exercises (20 min): The study assistant remains silently inside the room.	Two 30 min sessions (S1, S2: including measurements: 10 min)	VAS: before each session. PPG: continuous recording: S1, S2—20 min plus 5 min pre- and post-intervention (during rest).	acute pain; VM-HRV; BVP-A.	VAS: self-ratings of acute pain; PPG: sensor placed on the index finger of non-dominant hand: VM-HRV, BVP-A—cardiovascular, autonomic nervous system response	IG (Live MT): Higher levels of VM-HRV, stronger reductions in vascular sympathetic tone; recommended in pain and stress-related symptoms treatment
Warth 2015 [3] Germany	MT: live music–based relaxation exercises (by music therapists using voice, monochord). Short mindfulness exercise with soft monochord sounds; volume, dynamics, intensity adjusted to patient’s breathing; vocal improvisation (church modes), intensity gradually reduced (15 min); Patient reflected on listening experience (5 min)	CGa: listening to verbal relaxation exercise: excerpt from Mindfulness-Based Stress Reduction Program, played through headphones. The body scan meditation exercise: active control condition: Study assistant remained silent in the room	Two 30 min sessions (S1, S2; including measurements: 10 min) two days apart	VAS baseline, before, after S1, S2. PPG: baseline, throughout the session, intervals in milliseconds between successive heartbeats continuously recorded. Time segments of 5 min before and after the intervention. Mean amplitude of peripheral BVP-A calculated for the same time frame. EORTC QLQ-C15-PAL: at initial contact and at the end of S2.	relaxation, well-being, acute pain. Heart rate variability, sympathetic activity, quality of life	VAS: 0–10 self-ratings of relaxation, well-being, acute pain; PPG: HRV, continuously recorded intervals in milliseconds between successive heartbeats. Mean amplitude of peripheral BVP-A: high amplitude implies high blood flow in the fingertips and lower sympathetic activity; EORTC QLQ-C15-PAL	IG: improved subjective relaxation, well-being; increased high-frequency variations in HR; increased parasympathetic, reduced sympathetic modulations of cardiovascular activity; superior in Fatigue subscale; no pain reduction. Physiological data varied between individuals. QOL improved in both groups.

Abbreviations: BMGE, Brief Measure of Generativity and Ego-Integrity; BP, blood pressure; BVP-A, blood volume pulse amplitude; CGa, active control group; CGs, standard control group; CSQ-8, Client Satisfaction Questionnaire-8; EEG, electroencephalography; ELISA, enzyme-linked immunosorbent assay; EORTC QLQ-C15-PAL, European Organisation for Research and Treatment of Cancer Quality of Life Questionnaire-Core 15 Palliative; ESAS, Edmonton Symptom Assessment System; FACIT-Sp, Functional Assessment of Chronic Illness Therapy-Spiritual Well-Being; GCS, General Comfort Scale; HQOLI-R, Hospice Quality of Life Index-Revised; HR, heart rate; HRV, heart rate variability; IBI, inter-beat intervals; IG, intervention group; KPS, Karnofsky Performance Status; mHR, mean heart rate; MT, music therapy; MQOL-R, McGill Quality of Life Questionnaire Revised; NCCN DT, National Comprehensive Cancer Network Distress Thermometer; PPG, photoplethysmography; PPS, Palliative Performance Scale; PR, pulse rate; QOL, quality of life; RMSSD, root mean square of successive differences; RR, respiratory rate; S1, session 1; S2, session 2; S3, session 3; sAA, salivary alpha-amylase; sCort, salivary cortisol; SF-MPQ, Short Form McGill Pain Questionnaire; SOL, Song of Life; STAI, State-Trait Anxiety Inventory; VAS, Visual Analogue Scale; VAS-WBFPS, Visual Analogue Scale augmented with Wong–Baker Faces Pain Scale; VM-HRV, vagally mediated heart rate variability.

## Data Availability

All data relevant to this study have been included in the article and its Supplementary Materials. Additional data may be made available by the corresponding author upon reasonable request.

## Supplementary Materials

The following supporting information can be downloaded at: https://www.mdpi.com/article/10.3390/jcm15187057/s1, Supplementary Table S1: Deviations from the registered protocol; Supplementary Table S2: Detailed search strategy; Supplementary Table S3: Additional search strategies for Google Scholar, trial registries, and Cochrane CENTRAL; Supplementary Table S4: Summary of findings using the Grading of Recommendations Assessment, Development, and Evaluation (GRADE) system; Supplementary Table S5: Extracted numerical data and effect size calculations used in the meta-analysis; Supplementary Table S6: Outcome-specific risk-of-bias assessment using the RoB 2 tool with domain-level justifications; Supplementary Table S7: Methodological quality of the included 11 reports assessed using the Physiotherapy Evidence Database (PEDro) scale; Supplementary Figure S1: Forest plot of pain intensity after excluding the study at high risk of bias; Supplementary Figure S2: Descriptive presentation of pain intensity effects according to control condition; Supplementary Figure S3: Descriptive presentation of pain intensity effects according to number of sessions (≤3 vs. >3).
