# Supplementary material for: Music-Based Interventions in Advanced Cancer Palliative Care: A Systematic Review and Meta-Analysis of Randomized Controlled Trials Identified Within the Broader Field of Art-Based Interventions
Source: J Clin Med. 2026 Sep 11;15(18):7057. doi: 10.3390/jcm15187057 (PMC13607151; doi:10.3390/jcm15187057)
Supplement: Supplementary file 1 [file jcm-15-07057-s001.zip › jcm-4473790-supplementary.pdf]

Supplementary material

Title

Music-based interventions in advanced cancer palliative care: a systematic review and meta-analysis of randomized controlled trials identified within the broader field of art-based interventions

Authors

Agata Śmiłowska[1,2], Magdalena Stania[3], Małgorzata Gajda[4], Justyna Niesporek[4], Anna Polak[5], Jolanta Grabowska Markowska[2], Agnieszka Opala-Berdzik[6].

Supplementary Table S1. Deviations from the registered protocol

Review component	Registered protocol	Final review	Deviation/explanation
**Review title**	*Impact of art therapy interventions on advanced cancer patients under palliative care*	*Music-based interventions in advanced cancer palliative care: a systematic review and meta-analysis of randomized controlled trials identified within the broader field of art-based interventions*	The review title was revised to better reflect the scope and content of the completed review. The change did not alter the review question, population, interventions, comparators, or outcomes.
**Timing of the search**	At the time of registration on 27 January 2025, "Pilot work" and "Formal searching/study identification" were indicated as started, while screening, data extraction, risk-of-bias assessment, and data synthesis had not commenced.	Initial searches were conducted in PubMed (17 December 2024), Ovid MEDLINE (21 December 2024), and Embase (10 January 2025), shortly before protocol registration. An updated search of all databases was conducted in PubMed on 20 February 2026, Ovid MEDLINE on 17 February 2026, and Embase on 24 February 2026. Additional searches were conducted in ClinicalTrials.gov on 12 August 2026, WHO ICTRP and Cochrane CENTRAL on 13 August 2026, and Google Scholar on 16 August 2026.	According to PROSPERO registration guidance, "Pilot work" and "Formal searching/study identification" may have commenced before registration, provided that subsequent review stages have not started. PROSPERO therefore considers the protocol to have been prospectively registered. The final updated searches were conducted after registration and were used to ensure that the evidence base was current.
**Study design**	Only randomized controlled trials (RCTs) were eligible.	RCTs were included in the review.	**No deviation.**
**Population**	Adults with advanced cancer receiving palliative care; patients receiving causal cancer treatment,	Predominantly (≥95%) adults with advanced cancer receiving palliative care. Of 9 RCTs, 3 had a	3 of the 9 RCTs reported isolated cases of non-cancer participants (<5%). Given this very small proportion and the

Review component	Registered protocol	Final review	Deviation/explanation
	patients without palliative care, patients with non-cancer diagnoses, and caregivers/family members/healthcare staff were excluded.	small proportion of non-cancer patients,	limited number of eligible RCTs, excluding the entire RCT solely based on these isolated cases was considered disproportionate and could have resulted in an unnecessary loss of relevant evidence.
**Intervention**	Art-based interventions: music therapy, bibliotherapy, visual art therapy, drama therapy, poetry therapy, painting therapy, sculpting therapy, occupational therapy using art therapy, narrative art therapy, creative art therapy, expressive art therapy, dance therapy, dance-movement therapy, and movement-based art therapy..	Art-based interventions: music-based interventions, bibliotherapy, visual art therapy, drama therapy, poetry therapy, painting therapy, sculpting therapy, occupational therapy using art therapy, narrative art therapy, creative art therapy, expressive art therapy, dance therapy, dance-movement therapy, and movement-based art therapy..	"Music therapy" was refined to "music-based interventions" in the PICOS criteria to accurately reflect the range of music-related interventions evaluated in the included RCTs, some of which did not meet the formal definition of music therapy.
**Comparator/control**	Standard palliative care, defined as conventional medical interventions for symptom management, pain relief, and supportive care.	Studies with standard control or active control/comparator interventions were included (all receiving standard palliative care, defined as conventional medical interventions for symptom management, pain relief, and supportive care)	The eligibility criteria were broadened to include active comparator interventions in addition to standard palliative care. This change was made to allow inclusion of relevant randomized evidence in which art therapy was compared with another active intervention.
**Outcomes**	Main outcomes: pain, fatigue, sleep quality, anxiety, depression, and quality of life.	pain, fatigue, sleep quality, anxiety, depression, and quality of life.	Methodological clarification. ESAS-r was excluded as a measure of quality of life because it primarily assesses symptom burden rather than quality of life; therefore, only instruments specifically designed to assess quality of life, such as HQOLI-R, were included for this outcome.
**Effect measures**	Continuous outcomes were planned to be analyzed using MD and/or SMD with 95% CIs; dichotomous outcomes using RR or OR with 95% CIs.	For continuous outcomes, standardized mean differences (SMDs), expressed as Cohen's *d* with 95% CIs, were calculated. No small-sample correction was applied.	No substantive deviation. SMD was prespecified; Cohen's d was used because different scales were used to assess the same outcomes.
**Risk-of-bias assessment**	A tool such as JADAD, ROBINS, or PEDro was planned.	RoB 2 was used for both study-level and outcome-result-level risk-of-bias assessments.	RoB 2 was used to enable a rigorous, outcome-specific assessment of risk of bias.
**Data synthesis**	Meta-analysis was planned where appropriate, using fixed- or random-effects	A random-effects model was used for all meta-analyses, with the statistical	No substantive deviation. A random-effects model was used due to anticipated clinical and

Review component	Registered protocol	Final review	Deviation/explanation
	models depending on heterogeneity. Narrative synthesis was planned when meta-analysis was not possible.	approach further specified by reporting the τ^2 estimator and CI method.	methodological heterogeneity across studies.
**Subgroup analysis**	Subgroup analyses according to type of art therapy were planned, including music, bibliotherapy, visual art, drama, poetry, painting, sculpting, narrative, dance, and movement art therapy.	Exploratory subgroup analyses were instead performed according to control type and number of music-based intervention sessions, where data permitted.	The planned subgroup analysis by type of art therapy was not feasible because quantitative synthesis was restricted to music-based intervention.
**Sensitivity analysis**	Sensitivity analysis excluding RCTs with high risk of bias for one or more relevant domains was planned.	Minor refinement. Sensitivity analyses excluded studies at high risk of bias based on outcome-specific RoB 2 assessments.	Outcome-specific RoB 2 assessments were used to provide a more precise evaluation of the robustness of pooled results.
**Search update**	Searches were to be re-run before the final analysis.	The searches were rerun before the final analysis. PubMed was updated on 20 February 2026, Ovid MEDLINE on 17 February 2026, and Embase on 24 February 2026. Additional searches were subsequently conducted in ClinicalTrials.gov on 12 August 2026, WHO ICTRP and Cochrane CENTRAL on 13 August 2026, and Google Scholar on 16 August 2026.	The planned search update was conducted before the final analysis. In addition, the search was expanded to include ClinicalTrials.gov, WHO ICTRP, Cochrane CENTRAL, and Google Scholar, which were not specified in the registered protocol, in order to improve search comprehensiveness and identify additional ongoing, unpublished, or otherwise potentially relevant studies.

Supplementary Table S2. Detailed search strategy.

Last PubMed search: 20 February, 2026.

PubMed search strategy (link)

((complementary therapy[Title/Abstract]) OR (alternative therapy[Title/Abstract]) OR (bibliotherapy[Title/Abstract]) OR (narrative therapy[Title/Abstract]) OR (reminiscence bibliotherapy[Title/Abstract]) OR (narrative thinking[Title/Abstract]) OR (poetry therapy[Title/Abstract]) OR (music therapy[Title/Abstract]) OR (creative therapy[Title/Abstract]) OR (painting therapy[Title/Abstract]) OR (sculpting therapy[Title/Abstract]) OR (art therapy[Title/Abstract]) OR (healing art[Title/Abstract]) OR (occupational therapy[Title/Abstract]) OR (choreotherapy[Title/Abstract]) OR (expressive therapy[Title/Abstract]) OR (drama therapy[Title/Abstract]) OR (dance therapy[Title/Abstract]) OR (dance[Title/Abstract]) OR (dance-movement therapy[Title/Abstract]) OR (movement therapy[Title/Abstract]) OR (play therapy[Title/Abstract]) OR (creative art therapy[Title/Abstract]) OR (visual art therapy[Title/Abstract]) OR (narrative reminiscence therapy[Title/Abstract]) OR (movement psychotherapy[Title/Abstract])) AND ((cancer[Title/Abstract]) OR (tumor[Title/Abstract]) OR (neoplasms[Title/Abstract]) OR (terminal cancer[Title/Abstract]) OR (metastatic cancer[Title/Abstract]) OR (malignant[Title/Abstract]) OR (incurable disease[Title/Abstract])) AND ((palliative care[Title/Abstract]) OR (palliative medicine[Title/Abstract]) OR (hospice care[Title/Abstract]) OR (terminal care[Title/Abstract]) OR (end-of-life care[Title/Abstract]) OR (end of life care[Title/Abstract]) OR (hospice[Title/Abstract])) Filters: English

184 selected items

Last Embase search: 24 February, 2026.

No.	Query Results	Results	Date
#31.	#11 AND #30 AND [english]/lim	147	10 Jan 2025
#30.	#12 OR #13 OR #14 OR #15 OR #16 OR #17 OR #18 OR #19 OR #20 OR #21 OR #22 OR #23 OR #24 OR #25 OR #26 OR #27 OR #28 OR #29	63,536	10 Jan 2025
#29.	visual AND art AND therapy:ab,ti	1,446	10 Jan 2025
#28.	'play therapy':ab,ti	751	10 Jan 2025
#27.	'dancing':ab,ti	3,072	10 Jan 2025
#26.	'dance therapy':ab,ti	302	10 Jan 2025
#25.	'drama therapy':ab,ti	195	10 Jan 2025
#24.	expressive AND therapy:ab,ti	1,995	10 Jan 2025
#23.	'active music therapy':ab,ti	90	10 Jan 2025
#22.	'occupational therapy':ab,ti	19,103	10 Jan 2025
#21.	sculpting AND therapy:ab,ti	112	10 Jan 2025
#20.	painting AND therapy:ab,ti	1,038	10 Jan 2025
#19.	'painting':ab,ti	9,904	10 Jan 2025
#18.	creative AND therapy:ab,ti	16,964	10 Jan 2025
#17.	'music therapy':ab,ti	5,201	10 Jan 2025
#16.	'poetry therapy':ab,ti	66	10 Jan 2025
#15.	'narrative therapy':ab,ti	358	10 Jan 2025
#14.	'bibliotherapy':ab,ti	632	10 Jan 2025
#13.	'complementary therapy':ab,ti	3,094	10 Jan 2025
#12.	'art therapy':ab,ti	2,708	10 Jan 2025
#11.	#4 AND #10	23,513	10 Jan 2025
#10.	#5 OR #6 OR #7 OR #8 OR #9	73,982	10 Jan 2025
#9.	'end of life':ab,ti	48,084	10 Jan 2025
#8.	'terminal care':ab,ti	2,091	10 Jan 2025
#7.	'palliative therapy':ti,ab	3,460	10 Jan 2025
#6.	pallitive AND care	21	10 Jan 2025
#5.	'hospice':ti,ab	28,381	10 Jan 2025
#4.	#1 OR #2 OR #3	5,814,471	10 Jan 2025
#3.	'neoplasm':ab,ti	106,508	10 Jan 2025
#2.	'malignant neoplasm':ab,ti	9,116	10 Jan 2025
#1.	'cancer'/exp OR cancer:ab,ti	5,784,107	10 Jan 2025

No.	Query Results	Results	Date
#31.	#11 AND #30 AND [english]/lim	173	24 Feb 2026
#30.	#12 OR #13 OR #14 OR #15 OR #16 OR #17 OR #18 OR #19 OR #20 OR #21 OR #22 OR #23 OR #24 OR #25 OR #26 OR #27 OR #28 OR #29	74,784	24 Feb 2026
#29.	visual AND art AND therapy:ab,ti	1,732	24 Feb 2026
#28.	'play therapy':ab,ti	853	24 Feb 2026
#27.	'dancing':ab,ti	3,442	24 Feb 2026
#26.	'dance therapy':ab,ti	370	24 Feb 2026
#25.	'drama therapy':ab,ti	220	24 Feb 2026
#24.	expressive AND therapy:ab,ti	2,948	24 Feb 2026
#23.	'active music therapy':ab,ti	110	24 Feb 2026
#22.	'occupational therapy':ab,ti	21,718	24 Feb 2026
#21.	sculpting AND therapy:ab,ti	132	24 Feb 2026
#20.	painting AND therapy:ab,ti	1,244	24 Feb 2026
#19.	'painting':ab,ti	10,709	24 Feb 2026
#18.	creative AND therapy:ab,ti	21,058	24 Feb 2026
#17.	'music therapy':ab,ti	6,568	24 Feb 2026
#16.	'poetry therapy':ab,ti	76	24 Feb 2026
#15.	'narrative therapy':ab,ti	421	24 Feb 2026
#14.	'bibliotherapy':ab,ti	724	24 Feb 2026
#13.	'complementary therapy':ab,ti	3,671	24 Feb 2026
#12.	'art therapy':ab,ti	3,236	24 Feb 2026
#11.	#4 AND #10	26,411	24 Feb 2026
#10.	#5 OR #6 OR #7 OR #8 OR #9	82,706	24 Feb 2026
#9.	'end of life':ab,ti	54,128	24 Feb 2026
#8.	'terminal care':ab,ti	2,157	24 Feb 2026
#7.	'palliative therapy':ti,ab	3,725	24 Feb 2026
#6.	pallitive AND care	24	24 Feb 2026
#5.	'hospice':ti,ab	31,766	24 Feb 2026
#4.	#1 OR #2 OR #3	6,358,035	24 Feb 2026
#3.	'neoplasm':ab,ti	114,893	24 Feb 2026
#2.	'malignant neoplasm':ab,ti	9,922	24 Feb 2026
#1.	'cancer'/exp OR cancer:ab,ti	6,325,413	24 Feb 2026

Last MEDLINE Ovid search: 17 February, 2026

Ovid search strategy (link)

Ovid MEDLINE(R) ALL <1946 to February 17, 2026>

1 Cancer.mp. or Neoplasms/ or Malignant Neoplasms.mp. or Advanced cancer.mp. or End stage cancer.mp. or Tumor.mp. or Neoplasm Metastasis/ or Neoplasm Metastasis.mp. 4088659

2 Palliative Care/ or (Palliative Care or cancer palliative treatment).mp. or Palliative Medicine/ or Palliative Medicine.mp. or Terminal Care/ or terminal patient care.mp. or Hospice Care.mp. or (Care, Hospice or Hospice or Hospice Care).mp. 121122

3 Art Therapy/ or (Bibliotherapy/ or Narrative Therapy/ or Play Therapy/) or (Art Therapy or creative arts therapy).mp. or Complementary Therapies/ or Music Therapy/ or Music Therapy.mp. or Dance Therapy/ or Dance Therapy.mp. or Bibliotherapy.mp. or Drama Therapy.mp. or Dancing/ or Movement therapy.mp. or Poetry Therapy.mp. or Sculpture/ or Sculpture.mp. or Occupational Therapy/ or Creative therapy.mp. or Paintings/ or Painting.mp. or art painting.mp. 67382

4 1 and 2 and 3 358

5 limit 4 to english 332

Supplementary Table S3. Additional search strategies for Google Scholar, trial registries, and Cochrane CENTRAL.

Source	Search date	Search no.	Search strategy	Results retrieved	Records screened
Google Scholar	16 August 2026	1	"advanced cancer" AND "palliative care" AND "music therapy" AND ("randomized controlled trial" OR "randomised controlled trial")	~2030	300
Google Scholar	16 August 2026	2	"advanced cancer" AND "palliative care" AND "art therapy" AND ("randomized controlled trial" OR "randomised controlled trial")	~930	300
Google Scholar	16 August 2026	3	"advanced cancer" AND "palliative care" AND ("dance therapy" OR choreotherapy) AND ("randomized controlled trial" OR "randomised controlled trial")	~115	115
Google Scholar	16 August 2026	4	"advanced cancer" AND "palliative care" AND bibliotherapy AND ("randomized controlled trial" OR "randomised controlled trial")	~114	114
Google Scholar	16 August 2026	5	"advanced cancer" AND "palliative care" AND "poetry therapy" AND ("randomized controlled trial" OR "randomised controlled trial")	~34	34
Google Scholar	16 August 2026	6	"advanced cancer" AND "palliative care" AND "drama therapy" AND ("randomized controlled trial" OR "randomised controlled trial")	~51	51
Google Scholar	16 August 2026	7	"advanced cancer" AND "palliative care" AND "visual art therapy" AND ("randomized controlled trial" OR "randomised controlled trial")	17	17
Google Scholar	16 August 2026	8	"advanced cancer" AND "palliative care" AND "painting therapy" AND ("randomized controlled trial" OR "randomised controlled trial")	15	15
Google Scholar	16 August 2026	9	"advanced cancer" AND "palliative care" AND "creative art therapy" AND ("randomized controlled trial" OR "randomised controlled trial")	22	22
Google Scholar	16 August 2026	10	"advanced cancer" AND "palliative care" AND "sculpting therapy" AND ("randomized controlled trial" OR "randomised controlled trial")	0	0
Google Scholar	16 August 2026	11	"advanced cancer" AND "palliative care" AND "expressive arts therapy" AND ("randomized controlled trial" OR "randomised controlled trial")	~55	55
Google Scholar	16 August 2026	12	"advanced cancer" AND "palliative care" AND "healing art" AND ("randomized controlled trial" OR "randomised controlled trial")	~57	57
ClinicalTrials.gov	12 August 2026	-	("complementary therapy" OR "alternative therapy" OR bibliotherapy OR "narrative therapy" OR "reminiscence bibliotherapy" OR "narrative thinking" OR "poetry therapy" OR "music therapy" OR "creative therapy" OR "painting therapy" OR "sculpting therapy" OR "art therapy" OR "healing art" OR "occupational therapy" OR choreotherapy OR "expressive therapy" OR "drama therapy" OR "dance therapy" OR dance OR "dance-movement therapy" OR "movement therapy" OR "play therapy" OR "creative art therapy" OR "visual art therapy" OR "narrative reminiscence therapy" OR "movement psychotherapy") AND (cancer OR tumor OR neoplasms OR "terminal cancer" OR "metastatic cancer" OR malignant OR "incurable disease") AND ("palliative care" OR "palliative medicine" OR "hospice care" OR "terminal care" OR "end-of-life care" OR "end of life care" OR hospice)	126	126
WHO ICTRP	13 August 2026	-	(cancer OR tumor OR neoplasms OR "terminal cancer" OR "metastatic cancer" OR malignant OR "incurable disease" OR "palliative care" OR "palliative medicine" OR "hospice care" OR "terminal care" OR "end-of-life care" OR "end of life care" OR hospice) AND ("complementary therapy" OR "alternative therapy" OR bibliotherapy OR "narrative therapy" OR "reminiscence bibliotherapy" OR "narrative thinking" OR "poetry therapy" OR "music therapy" OR "creative therapy" OR "painting therapy" OR "sculpting therapy" OR "art therapy" OR "healing art" OR "occupational therapy" OR choreotherapy OR "expressive therapy" OR "drama therapy" OR "dance therapy" OR dance OR "dance-movement therapy" OR "movement therapy" OR "play therapy" OR "creative art therapy" OR "visual art therapy" OR "narrative reminiscence therapy" OR "movement psychotherapy")	343 records (331 trials)	331
Cochrane CENTRAL	13 August 2026	-	#1 (cancer OR tumor OR neoplasms OR "terminal cancer" OR "metastatic cancer" OR malignant OR "incurable disease")<br>#2 ("palliative care" OR "palliative medicine" OR "hospice care" OR "terminal care" OR "end-of-life care" OR "end of life care" OR hospice)<br>#3 ("complementary therapy" OR "alternative therapy" OR bibliotherapy OR "narrative therapy" OR "reminiscence bibliotherapy" OR "narrative thinking" OR "poetry therapy" OR "music therapy" OR "creative therapy" OR "painting therapy" OR "sculpting therapy" OR "art therapy" OR "healing art" OR "occupational therapy" OR choreotherapy OR "expressive therapy" OR "drama therapy" OR "dance therapy" OR dance OR "dance-movement therapy" OR "movement therapy" OR "play therapy" OR "creative art therapy" OR "visual art therapy" OR "narrative reminiscence therapy" OR "movement psychotherapy")<br>#4 #1 AND #2 AND #3	87	87

Supplementary Table S4. Summary of findings using the Grading of Recommendations Assessment, Development, and Evaluation (GRADE) system.

Certainty assessment							№ of patients		Effect		Certainty	Importance
**№ of studies**	**Study design**	**Risk of bias**	**Inconsistency**	**Indirectness**	**Imprecision**	**Other considerations**	**Music-based interventions**	**control**	**Relative (95% CI)**	**Absolute (95% CI)**		
**Pain intensity**												
3	randomised trials	very serious[a]	serious[b]	not serious	serious[c]	none	112	112	-	SMD **0.51 SD lower** (0.98 lower to 0.03 lower)	⊕◯◯◯ Very low[a,b,c]	NOT IMPORTANT
**Quality of life_HQOLI-R**												
2	randomised trials	very serious[a]	not serious	not serious	very serious[d]	none	97	71	-	SMD **0.92 SD higher** (0.59 higher to 1.24 higher)	⊕◯◯◯ Very low[a,d]	NOT IMPORTANT

CI: confidence interval; **MD:** mean difference; **SMD:** standardised mean difference; Explanations: a. High risk of bias due to concerns regarding randomization, deviations from intended interventions, missing outcome data, and outcome measurement. b. Significant heterogeneity was observed: $I^2=67.69\%$, $\tau^2=0.119$; $p=0.045$; c. Small sample size; d. Small sample size, wide confidence intervals around estimates of effect.

Supplementary Table S5. Extracted numerical data and effect size calculations used in the meta-analysis.

Study	Outcome	Time point	Cancer-only sample n	Intervention group n	Intervention group mean±SD	Control group n	Control group mean±SD	Effect measure	Effect size
Düzgün and Karadakovan, 2024	Pain intensity<br><br>Patient Follow-Up Form – pain score (scale not clearly specified)	5 min after the last (6th) intervention session	Cancer-only population<br><br>60	30	3.77±1.73	30	5.20±1.71	Cohen's d	-0.83
Valero-Cantero et al., 2024	Pain intensity<br><br>Edmonton Symptom Assessment System (ESAS)	immediately after the 7-day intervention	Cancer-only population<br><br>80	40	3.38±3.09	40	5.38±2.83	Cohen's d	-0.68
Warth et al., 2015	Pain intensity<br><br>Visual Analog Scale (VAS)	immediately after the last (2nd) intervention session	Mixed population – 84; non-cancer patients, n = 2	42	2.45±2.10	42	2.57±2.16	Cohen's d	-0.06
Hilliard, 2003	Quality of life<br><br>HQOLI-R Questionnaire	immediately after the second intervention	Cancer-only population 80	40	214.6±35.3	40	177.9±36.8	Cohen's d	1.02
Liao et al., 2013	Quality of life<br><br>HQOLI-R Questionnaire	after completion of the 3-week intervention	Cancer-only population 88	57	56.10±8.65	31	48.50±10.37	Cohen's d	0.82

Supplementary Table S6. Outcome-specific risk-of-bias assessment using the RoB 2 tool with domain-level justifications.

Study	Outcome	Time point	D1	D2	D3	D4	D5	Overall RoB
Düzgün and Karadakovan, 2024	Pain intensity	5 min after the last (6th) intervention session	Some concerns	High risk	High risk	High risk	Some concerns	High risk
			Allocation concealment and sequence generation were insufficiently described.	Participants were not blinded to treatment allocation, and pain was a subjective outcome potentially influenced by awareness of the intervention.	Only 60/78 randomized participants contributed data; missingness related to the true pain outcome cannot be excluded.	Pain was self-reported and participants were aware of intervention assignment, which could have influenced ratings.	Multiple measurements/analyses were possible without a reported prespecified protocol	
Valero-Cantero et al., 2024	Pain intensity	immediately after the 7-day intervention	Low risk	Some concerns	Low risk	Some concerns	Low risk	Some concerns
			Opaque, sealed, shuffled and numbered envelopes were used for random allocation, with no important baseline imbalances.	Participants were aware of the type of audio received, and intervention-related deviations cannot be fully excluded.	Outcome data were available for all 80 randomized participants with no loss to follow-up.	Although outcome assessors were blinded, pain was self-reported and participants were aware of the type of audio received, which could have influenced pain ratings.	The trial was prospectively registered, and ESAS assessment before and immediately after the intervention was prespecified.	
Warth et al., 2015	Pain intensity	immediately after the last (2nd) intervention session	Low risk	Some concerns	Low risk	Some concerns	Low risk	Some concerns
			Computerized block randomization was used, with allocation implemented through sealed, sequentially numbered envelopes.	Participants and therapists were aware of the intervention received; although ITT analysis was used, intervention-related deviations cannot be fully excluded.	Reasons for missing data were documented, and ITT analysis with imputation was performed; complete-case sensitivity analysis showed that the pain result was robust to missing-data handling.	Pain was self-reported using VAS; participants were blinded to the study hypothesis but were aware of the intervention received.	Pain and post-session assessment were prespecified, and a study protocol was published before the trial report.	
Hilliard, 2003	Quality of life	immediately after the second intervention	Some concerns	Low risk	Low risk	High risk	Some concerns	High risk
			Participants were reported as randomly assigned, but the method	Participants and intervention providers were aware of	All 80 randomized participants completed at least two QoL	QoL was self-reported, participants were aware of treatment allocation,	Multiple QoL assessments were conducted, and no	

			of sequence generation and allocation concealment was not described.	intervention assignment; however, no deviations from the intended interventions arising because of the trial context were reported.	assessments and contributed data to the analysis.	and the HQOLI-R was administered with assistance from unblinded music therapists or counselors.	prespecified protocol or analysis plan was reported to verify the selection of the analyzed result.	
Liao et al., 2013	Quality of life	after completion of the 3-week intervention	Low risk	Some concerns	Some concerns	Some concerns	Some concerns	Some concerns
			Random sequence generation was computer-based, and allocation was concealed using sealed opaque envelopes.	Participants were reportedly blinded, but researchers knew the intervention assignment; intervention-related deviations cannot be fully excluded.	Missing outcome data were limited (14/160); reasons for attrition were reported, but some losses were due to disease deterioration, and a relationship between missingness and QoL cannot be excluded.	QoL was self-reported; although participants were reported as blinded, researchers knew allocation, and the success of participant blinding is uncertain because the interventions were clearly different.	QoL was measured at multiple time points, and no prespecified protocol or statistical analysis plan was reported to verify that the analyzed time point/result was selected independently of the findings.	

D1: bias arising from the randomization process; **D2**: bias due to deviations from intended interventions; **D3**: bias due to missing outcome data; **D4**: bias in measurement of the outcome; **D5**: bias in selection of the reported result; **RoB**, risk of bias.

Supplementary Table S7. Methodological quality of the included 11 reports assessed using the Physiotherapy Evidence Database (PEDro) scale.

Reference	Eligibility criteria specified	Subjects randomly allocated to groups	Allocation concealed	Groups similar at baseline	Blinding of all subjects	Blinding of all therapists	Blinding assessors	> 85% follow up	Intention-to-treat analysis	Between-group statistical comparison	Point and variability measures	Score *
Düzgün 2024	+	+	-	+	-	-	-	-	-	+	+	**4/10**
Fernando 2019	+	+	-	+	-	-	-	+	+	+	+	**6/10**
Hilliard 2003	+	+	-	+	-	-	-	+	+	+	+	**6/10**
Horne-Thompson 2008	+	+	+	+	+	-	+	-	-	+	+	**7/10**
Koehler 2022	+	+	+	+	+	-	-	+	+	+	+	**8/10**
Liao 2013	+	+	+	+	+	-	-	+	-	+	+	**7/10**
Ramirez 2018	+	+	-	-	+	-	-	+	+	+	+	**6/10**
Valero-Cantero 2024	+	+	+	+	+	-	+	+	+	+	+	**9/10**
Warth 2021	+	+	+	+	+	-	-	-	+	+	+	**7/10**
Warth 2016	+	+	+	+	-	-	-	+	-	+	+	**6/10**
Warth 2015	+	+	+	+	-	-	-	+	+	+	+	**7/10**

**Eligibility criteria* item is not included in PEDro score calculations

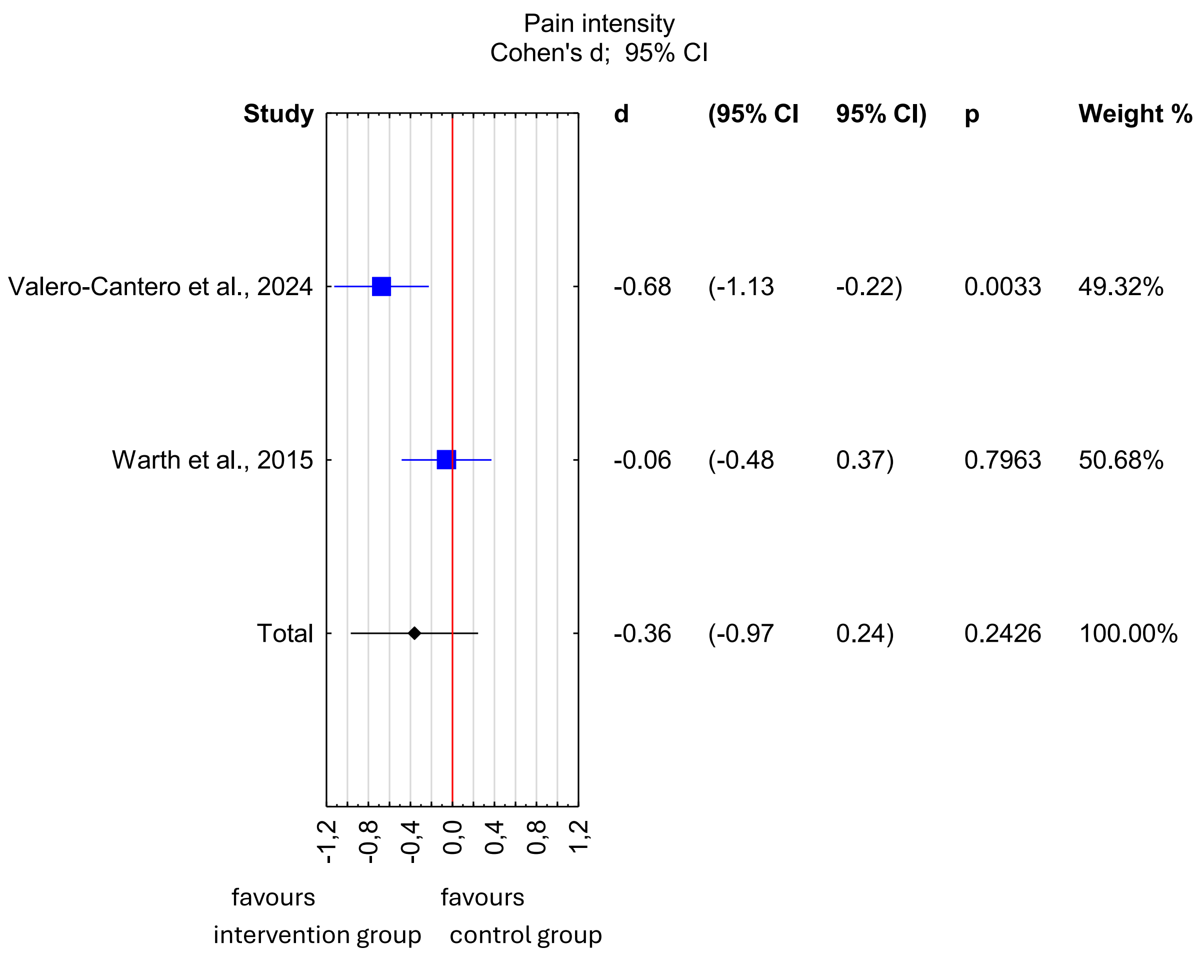

Supplementary Figure S1 Forest plot of pain intensity after excluding the study at high risk of bias

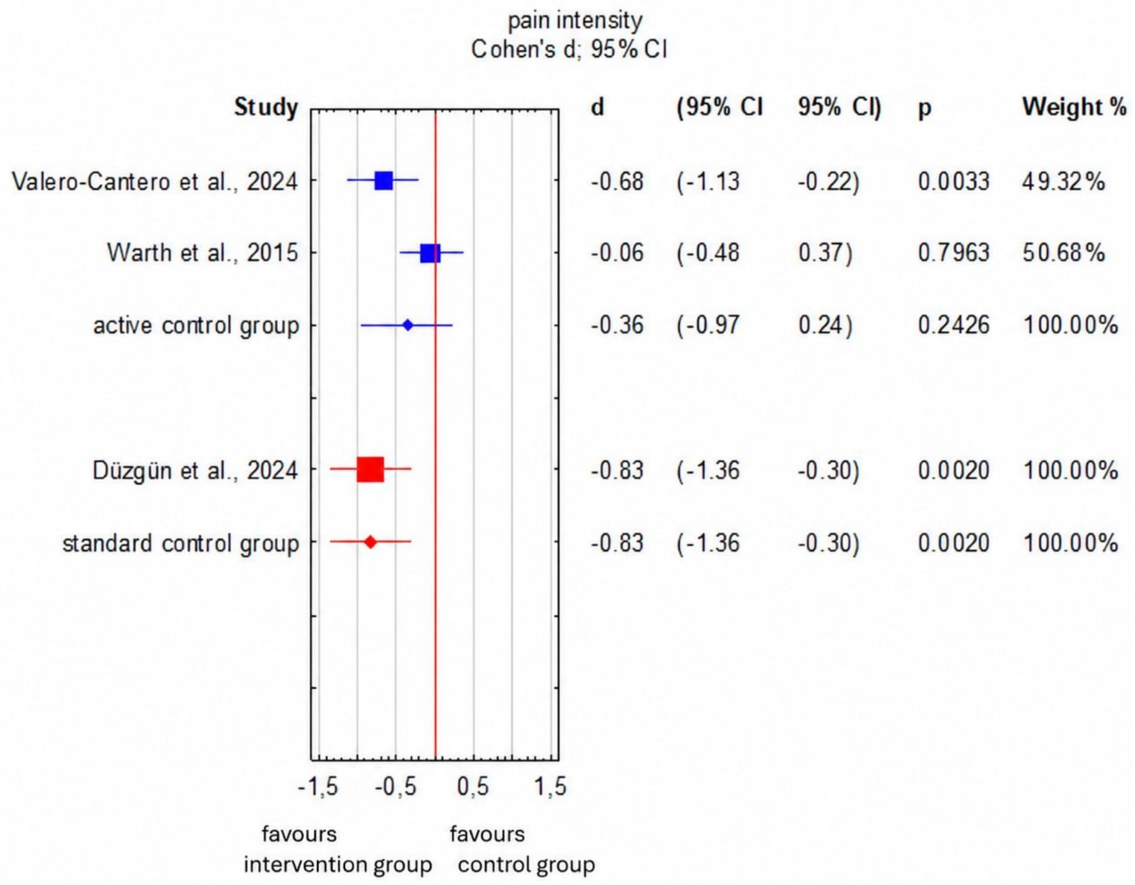

Supplementary Figure S2. Descriptive presentation of pain intensity effects according to control condition.

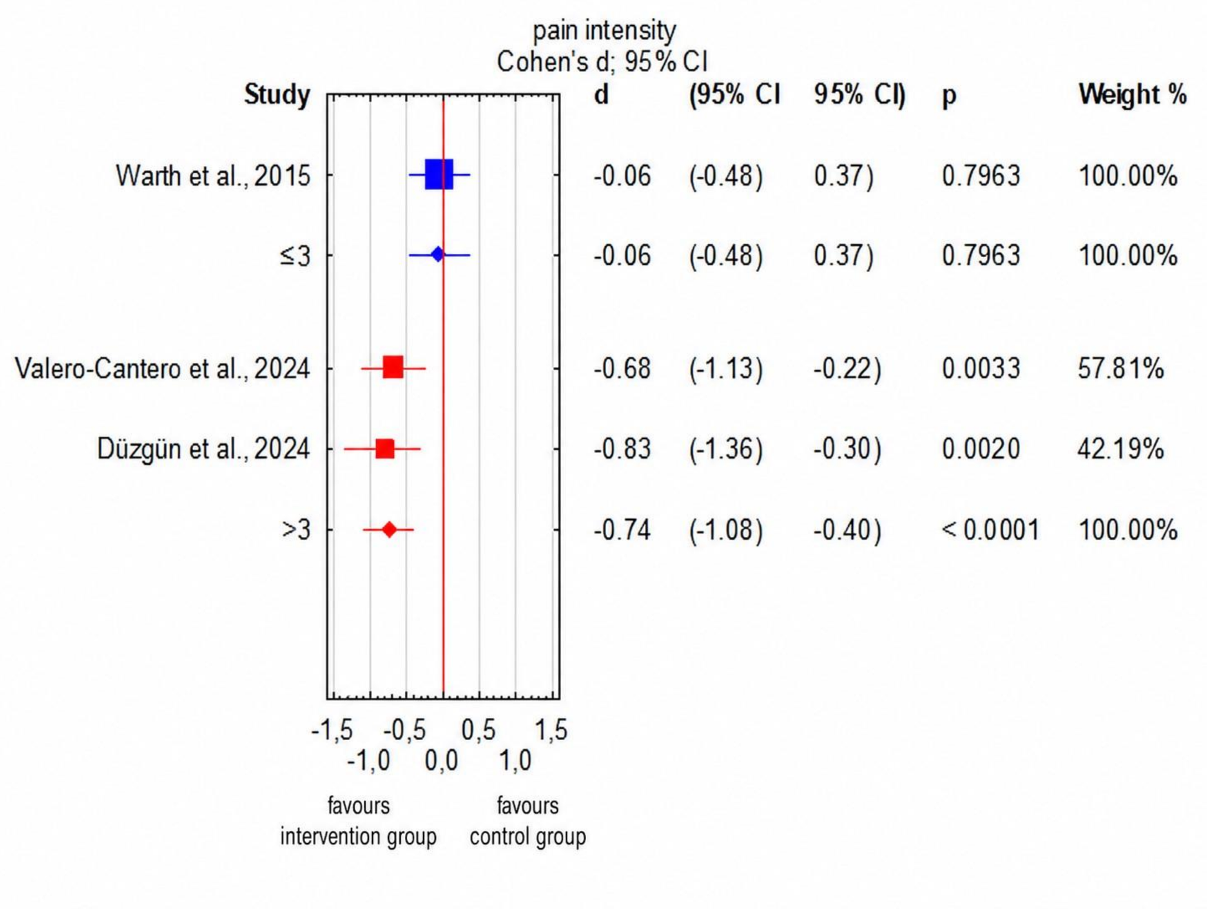

Supplementary Figure S3. Descriptive presentation of pain intensity effects according to number of sessions (≤3 vs. >3).
